# Supplementary material for: Quantifying the impact of a broadly protective sarbecovirus vaccine in a future SARS-X pandemic
Source: Nat Commun. 2025 Sep 26;16:8495. doi: 10.1038/s41467-025-63399-x (PMC12475435; doi:10.1038/s41467-025-63399-x)
Supplement: Supplementary file 1 — Supplementary Information [file 41467_2025_63399_MOESM1_ESM.pdf]

Quantifying the impact of a broadly protective sarbecovirus vaccine in a future SARS-X pandemic

Supplementary Information:

1. **Supplementary Methods: Branching Process Framework**

2. **Supplementary Methods: Dynamical Compartmental Modelling Framework**

3. **Supplementary Methods: Dynamical Compartmental Modelling Framework Equations**

4. **Supplementary Figures and Results**

5. **References**

Supplementary Information

1. Supplementary Methods: Branching Process Framework

1.1 Overview of Stochastic Branching Process Modelling Framework

We extended a stochastic branching-process modelling framework initially developed to simulate SARS-CoV-2 transmission and control through contact tracing *(1, 2)*, and use this framework to explore a number of different BPSV vaccination strategies aimed at early stage containment and suppression of outbreaks. We explore this for two pathogen "archetypes" – the first pathogen archetype has properties similar to SARS-CoV-1, and is characterised by a long generation time, a limited degree of pre-symptomatic transmission, low proportion of asymptomatic infections and high disease severity. The second is SARS-CoV-2, characterised by a shorter generation time, high proportion pre-symptomatic transmission, moderate proportion of asymptomatic infections and low disease severity. For both pathogens, we vary the basic reproduction number across a range of values. We used this framework to simulate the impact of two different vaccine-based containment strategies utilising the BPSV. These were ring-vaccination (where detection of symptomatic cases triggers reactive vaccination of all contacts of that case) and spatially targeted vaccination (where hospitalised cases trigger a vaccination campaign that seeks to vaccinate all individuals in a defined geographic area). Below, we describe the two strategies in mathematical and technical terms. Full details of the code implementing these models can be found at https://github.com/mrc-ide/diseaseX_modelling/tree/main/functions.

1.2 Modelling Ring-Vaccination Campaigns

Overview: Within the ring-vaccination framework, following identification of the pathogen (assumed here to occur 21 days following a pathogen spillover event with 5 individuals initially infected), detection of a new symptomatic infection triggers reactive vaccination of all their contacts, which occurs after a delay of 2 days, reflecting the time to identify, notify and administer vaccination to contacts. Vaccinated individuals go on to develop immunological protection following vaccination at a delay of d_V days. Below, we outline the steps that follow within the simulation that describe how ring-vaccination is implemented.

Step 1: Selecting the next infection to process and generating potential secondary infections

The simulation framework is "asynchronous" and processes the next infection in absolute time order – we therefore select the earliest infection whose secondary cases have not yet been generated through taking a draw from the offspring distribution. This "index" infection anchors all subsequent timings in the current iteration.

For this index infection, the initial, potential number of secondary infections (before accounting for isolation and/or ring-vaccination) is drawn from the offspring distribution:

$$N_{\text{inf}} \sim Poisson(R_0)$$

where R_0 is the basic reproduction number i.e. the average number of secondary infections produced by a single primary infection. If the index was itself a *breakthrough* case – i.e. a breakthrough infection occurring in an already vaccinated individual, we assume that BPSV vaccination renders breakthrough infections less infectious and hence these individuals have reduced transmissibility. The total number of secondary infections produced is drawn from the following modified offspring distribution:

$$N_{\text{inf}} \sim Poisson(R_0(1-V_T))$$

where V_T is the BPSV efficacy against onwards transmission in vaccinated individuals with breakthrough infections.

Step 2: Determining the index case symptom status and timing of events in the disease course

For each of the N_{inf} potential secondary infections generated in **Step 1**, we draw a time of infection (relative to the time of the index infection) from the generation time distribution, such that the time of infection for each secondary infection τ_{inf} is given by:

$$\tau_{\text{inf}} \sim Gamma(\alpha_{Gt}, \beta_{Gt})$$

A Bernoulli draw with probability p_{asymp} (the probability that an infection is asymptomatic) determines whether the index case is asymptomatic. If symptoms occur, an incubation period τ_{inc} is drawn from the infection-to-symptom-onset distribution, such that $\tau_{\text{inc}} \sim Gamma(\alpha_{inc}, \beta_{inc})$. The absolute time of symptom onset is then calculated as $t_{\text{onset}} = t_{\text{inf}} + \tau_{\text{inc}}$, where t_{inf} is the absolute time at which the *index* infection was infected. What follows are a series of steps determining whether these N_{inf} potential secondary infections will be successfully averted by control measures (namely isolation/quarantine and ring-vaccination), or whether they will still happen despite these control measures.

Step 3: Determining whether and when the index case isolates, and the impact this has on secondary infections

Within the simulation framework, we assume that some fraction of infected individuals isolate and quarantine in response to symptoms. Specifically, isolation can be triggered via two distinct pathways:

1. **Contact-tracing isolation.** If the index case's *infector* had symptoms, public-health tracing reaches the index (infected by the index case's *infector*) and successfully leads to isolation/quarantine with probability $p_{iso,ct}$ (which is the probability inputted to a Bernoulli draw determining whether the index isolates successfully). If the index successfully quarantines and isolates, it does so with a delay (relative to the time of symptoms in the index case's *infector*) of $\tau_{iso,ct}$, which is drawn from the quarantine-delay distribution $\tau_{\text{iso,ct}} \sim Gamma(\alpha_{iso,ct}, \beta_{iso,ct})$. The absolute start of isolation is therefore given by

$$t_{iso,ct} = t_{onset,infector} + \tau_{\text{iso,ct}}$$

   where $t_{onset,infector}$ is the absolute time of symptom onset in the index case's infector.

2. **Self-initiated isolation.** If the index case's *infector* was asymptomatic, no contact tracing isolation can occur. However, if the index case itself becomes symptomatic, the index self-isolates with probability $p_{iso,symp}$ (which is the probability inputted to a Bernoulli draw determining whether the index isolates successfully). If the index successfully quarantines and isolates, it does so with a delay $\tau_{iso,symp}$, which is drawn from the quarantine-delay distribution $\tau_{\text{iso,symp}} \sim Gamma(\alpha_{iso,symp}, \beta_{iso,symp})$. The absolute start of isolation is therefore given by

$$t_{iso,symp} = t_{onset,index} + \tau_{\text{iso,symp}}$$

   where $t_{onset,index}$ is the absolute time of symptom onset in the index case.

If neither the infector nor the index is symptomatic, no trigger for isolation and quarantine occurs and thus is assumed to not occur. If both triggers are met (i.e. both the infector and index case are symptomatic) and successful isolation could occur through either route, the earlier route (i.e. the earlier of $t_{iso,symp}$ and $t_{iso,ct}$) is selected)

For each of the N_{inf} secondary infections j labelled $j = 1, \ldots, N_{inf}$, we then assess whether the timing of infection j occurs *after* the moment the index case goes into isolation and thus whether that infection is available to be averted by isolation. Let t_j^{inf} be the absolute calendar time at which infection j would take place and t_{iso} the absolute start-time of the index case's isolation, obtained from either *contact-tracing isolation (* $t_{iso,ct}$ *) or self-initiated isolation* ($t_{iso,symp}$). If no isolation is triggered, we set $t_{iso} = +\infty$. Infections are then pruned according to the following pruning rule:

$$\text{If } t_j^{\inf} \geq t_{index}^{iso} \rightarrow \text{infection } j \text{ is cancelled with probability } E_Q$$

where E_Q is the **quarantine efficacy** i.e. the proportional reduction in transmission from an infectious individuals that occurs following their isolation. For each of the j secondary infections, we therefore draw:

$$I_j \sim Bernoulli(1 - E_Q)$$

and retain infection j only if $I_j = 1$.. When $t_j^{\inf} < t_{index}^{iso}$ (i.e. infectious exposure precedes isolation) the infection automatically survives, i.e. $I_j = 1$. This rule is applied identically whether isolation arose through **contact tracing** or through **self-initiated symptom-based isolation**; the two pathways differ only in how they set the value of t_{index}^{iso}. After all $N_{\inf}$ candidates have been evaluated, the surviving infections, with total count denoted N_{inf}^* are taken forward to the ring-vaccination step. Any infections cancelled here permanently exit the transmission tree and do not contribute further to transmission.

Step 4: Scheduling the timing of a ring-vaccination campaign around the index case
If the index is symptomatic and the vaccine is already available (≥ 21 days post-spill-over), all listed contacts are offered vaccine at *logistical delay* $\delta_{\log istical} = 2$ days after the index's symptom onset:

$$t_{\text{vax}} = t_{\text{onset,index}} + \delta_{\text{logistical}}$$

Following vaccination, protection develops following a delay such that:

$$t_{\text{prot}} = t_{\text{vax}} + d_V$$

where d_V is the delay from vaccination to protective immunity.

Step 5: Applying ring-vaccination logic to each putative offspring
This step is executed only when (a) at least one offspring remains after pruning of infections due to isolation/quarantine (i.e. $N_{inf}^* > 0$), (b) the index is symptomatic, and (c) the calendar time exceeds the time from which the vaccine is available. For each available secondary infection generated by the index infection following isolation infections j labelled $j = 1, ..., N_{inf}^*$:

- If $t_{inf}^j > t_{vax}$ (infection j occurs after secondary infections can be vaccinated), the contact (representing infection j and who would otherwise be infected) accepts the vaccine with probability c_{cov}. Further, if $t_{inf}^j > t_{prot}$, then protection from vaccination arises in infection j before they would otherwise be infected, and the infection is averted with probability V_i (vaccine efficacy against infection). Averted infections are deleted from the simulation. Instances where vaccination fails to avert infection become *vaccinated breakthroughs*; they proceed but will later transmit at the reduced rate $(1 - V_T)$. Infections who decline vaccination remain unaltered and are retained within the simulation.

- If $t_{inf}^j < t_{vax}$ (the timing of infection j precedes ring-vaccination), the infection is not averted by vaccination and proceeds unaltered.

During this vaccination campaign, a number of different outcomes are therefore possible for the contacts of index cases that would otherwise be infected:

1) The individual is successfully vaccinated during the ring-vaccination campaign, protection arises before they would otherwise be infected, and their infection is successfully averted **(Outcome A)**.

2) The individual is successfully vaccinated during the ring-vaccination campaign, protection arises before they would otherwise be infected, but this protection fails to avert their infection **(Outcome B)**.
3) The individual is successfully vaccinated during the ring-vaccination campaign but infected before vaccine-derived protection arises **(Outcome C)**.
4) The individual is infected before the ring-vaccination campaign can be carried out **(Outcome D)**.
5) The individual is not identified by the ring-vaccination strategy and so is not vaccinated (assumed here to be 20% of contacts) **(Outcome E)**.

Below, we define formally the mathematical conditions under which each of these outcomes arises:

Outcome	Verbal description	Mathematical condition
**A. Averted infection**	Contact is vaccinated, protection arises in time, and infection is prevented.	Vaccinated **and** $t_{inf}^{j} > t_{prot}$ **and** Bernoulli success with probability V_i
**B. Breakthrough (protected)**	Vaccinated, protection arises in time, but infection nevertheless occurs.	Vaccinated **and** $t_{inf}^{j} > t_{prot}$ **and** Bernoulli failure with probability V_i.
**C. Vaccinated too late**	Vaccinated, but infection precedes protection from vaccination developing.	Vaccinated **and** $t_{inf}^{j} < t_{prot}$
**D. Infection before campaign**	Infection occurs before vaccination teams arrive.	$t_{inf}^{j} < 21$ (start of vaccine availability)
**E. Missed contact/not vaccinated**	Contact is not offered or refuses vaccine (20% of contacts by assumption).	Bernoulli failure with probability $1 - c_{\text{cov}}$

where V_i is the **vaccine efficacy against infection** (probability that a fully protected individual *escapes* infection upon exposure), and c_{cov} is the **ring-vaccination coverage** (proportion of listed contacts successfully reached and offered vaccine).

Outcomes A and B can only occur once the pathogen has been detected (i.e. following a calendar time ≥ 21 days) and only if the triggering index case was symptomatic; symptomatic carriers are assumed to remain undetected and therefore do **not** initiate a ring. After all offspring are processed, those not prevented by vaccination remain in the epidemic tree; the susceptible pool is reduced accordingly, and the algorithm returns to step (i) to process the next earliest infection (either until 10,000 infections are reached or there are no longer any infections to select).

Box S1: Pseudocode Description of the Ring-Vaccination Algorithm

```
Input:  N (population size), I0 (initial seed infections), epidemic_threshold, offspring distribution,
        natural-history parameters, and intervention parameters (vaccination, quarantine)

Initialise susceptible pool: S ← N − I0. Create I0 seed cases (IDs 1 … I0).
while total_cases < check_final__and S > 0 do
    i ← select earliest unsimulated infection

  # Generate secondary infections and their timings
    Draw Nᵢ offspring from offspring_distribution(R0)
    Draw natural-history times for i (asymptomatic, incubation period, etc.)
    Draw infection times for offspring from generation time distribution.

  # Implement Quarantine/Isolation
    Determine quarantine outcome for i based on asymptomatic flag
    if i isolates then
        Cull offspring whose infection time ≥ t_isolation with efficacy E_Q
    end if

  # Implement Ring Vaccination
    if i is symptomatic and current_time ≥ t_vax_start
        for each potential offspring j do
            Determine vaccination status (coverage c_vax) and timing of protection developing
            if vaccination prevents infection
                discard j
            else
                if vaccination occurred & protection active then
                    mark j as breakthrough (transmissibility × (1 − V_T))
                end if
            end if
        end for
    end if
    Insert retained offspring into dataframe
end while
Return complete infection-history dataframe
```

1.3 Modelling Spatially-Targeted Vaccination Strategies

Overview: To model the spatially targeted vaccination strategy, we modify the branching process such that each new infection is imbued with a set of geographical coordinates (their "home address") $\{x_i, y_i\}$. Following pathogen spill-over, a **geographically targeted** vaccination campaign is launched as soon as the cumulative number of hospitalised cases reaches a detection threshold H_{Thresh}. The infection that meets this threshold (the "trigger" case) defines the campaign's epicentre. Everyone located within a fixed radius r_{vax} of the trigger's home coordinates is eligible for vaccination. Because health authorities must confirm the hospital admission, mobilise teams, and reach the affected zone, vaccination begins after a logistical delay of 2 days following the timing of hospitalisation in the "trigger" case. As before, each vaccinated individual then requires an additional d_V days to develop protective immunity. Below we describe in detail the simulation steps followed to implement the spatial vaccination strategy. Many of these are similar to those implemented for ring-vaccination (described above) and we therefore focus on the steps that meaningfully differ.

Step 1: Selecting the next infection to process and generating potential secondary infections
As before, the initial number of secondary infections (before accounting for isolation and/or ring-vaccination) is drawn from the offspring distribution:

$$N_{\text{inf}} \sim Poisson(R_0) \text{ or } N_{\text{inf}} \sim Poisson(R_0(1-V_T))$$

depending on the vaccination status of the index infection, and where R_0 is (as before) the basic reproduction number, and where V_T is the BPSV efficacy against onwards transmission in vaccinated individuals with breakthrough infections. Additionally however, each secondary infection is imbued with geographical coordinates, with these coordinates depend on the coordinates of the infector and a spatial kernel, which is a distribution describing the probability of the home addresses of two directly linked infections being separated by a certain amount of distance. Together with a direction, these factors determine the "home address" of newly generated infections. Specifically, for each of the N_{inf} secondary infections j labelled $j = 1,...,N_{inf}$, we generate spatial coordinates for each through the following 3 steps:

- Draw $m = KN_{inf}$ preliminary radial distances $r^*_{j,1}, r^*_{j,2},...,r^*_{j,m} \sim k(r)$ where $k(r)$ is a one-dimensional distance kernel and K is an oversampling factor.
- Importance resample these radial distances to derive final radial distances. The probability that a neighbour lies in the annulus $(r, r+dr)$ (where dr is some small value) is proportional to its area $2\pi r dr$. An isotropic (i.e. direction and distance preserving) two-dimensional kernel must have radial density $g(r) \propto rk(r)$. To achieve this (i.e. convert the one-dimensional distance kernel $k(r)$ to an isotropic two-dimensional kernel $g(r)$), we select N_{inf} distances ($r_{j,1}, r_{j,2},...,r_{j,Ninf}$)from the candidate set $r^*_{j,1}, r^*_{j,2},...,r^*_{j,m}$ with the probability:

$$\Pr(r_j = r^*_{j,l}) = \frac{r^*_{j,l}}{\sum_{l'=1}^{m} r^*_{j,l'}}, l = 1,...,m$$

  which up-weights more distant candidates in proportion to annular area. This prevents an erroneously high density of offspring accumulating near the parent when the same values are spread uniformly over angles θ (drawn in the next step), and which would be consistent with the 1-dimension distance kernel, but not when extended to the 2-dimensional case. Without this correction, distances drawn from the one-dimensional kernel would pack a disproportionate share of points into the small inner rings and create an artificial crowding around the parent—an artefact of extending a 1-dimensional kernel to a 2-dimensional setting.

- Draw a direction $\theta_j \sim Uniform(0, 2\pi)$. If $\{x_{parent}, j_{parent}\}$ is the "home address" of the index infection, then the "home address" of each secondary infection is then calculated as:

$$x_j = x_{\text{parent}} + r_j \cos\theta_j$$
$$y_j = y_{\text{parent}} + r_j \sin\theta_j$$

We note that the exact spatial kernel will depend on specific features of the setting, population and pathogen being considered. We therefore considered a simplified case whereby instead of explicitly specifying a spatial kernel in absolute terms, we describe it relative to the radius of the spatial vaccination campaign that is implemented (see the detailed information about model parameterisation below).

Step 2: Determining the index case symptom status and timing of events in the disease course

Events in the disease course and natural history of disease are handled as in the ring-vaccination model. However, because hospitalisations are the trigger for initiation of the spatially targeted vaccination campaign, we explicitly model hospitalisations. A Bernoulli draw with probability p_{hosp} (the probability that an infection is hospitalised) determines whether each infection generated by the index case will go on to be hospitalised i.e. $H_j \sim \text{Bernoulli}(p_{\text{hosp}})$. If they are hospitalised ($H_j = 1$), we also draw the time between being infected and being admitted to hospital, which is given by $\tau_{hosp} \sim Gamma(\alpha_{hosp}, \beta_{hosp})$, and record the absolute admission time $t_j^{\text{adm}} = t_j^{\text{inf}} + \tau_j^{\text{hosp}}$. We track the cumulative number of hospitalisations H_{Tot} up to calendar time t as:

$$H(t) = \sum_{j=1}^{N(t)} H_j \mathbf{1}(t_j^{\text{adm}} \le t)$$

where $N(t)$ is the number of infections that have occurred by time t and $\mathbf{1}(t_j^{\text{adm}} \le t)$ is the indicator function. The spatial-vaccination campaign is triggered when $H(t)$ first reaches the pre-specified threshold H_{Thresh}.

Step 3: Determining whether and when the index case isolates, and the impact this has on secondary infections

Events relating to isolation and quarantine are handled as in the ring-vaccination model.

Step 4: Scheduling the timing of the spatially targeted vaccination campaign around the trigger infection

In contrast to the ring-vaccination strategy, spatial vaccination is not reactive to every case. Instead, it begins the moment the cumulative number of hospitalised cases $H(t)$ reaches the pre-set threshold H_{Thresh}. Let the infection that meets this threshold be indexed by

$$i = H_{Thresh}$$

with an infection time and spatial coordinates:

$$t_{\text{trigger}} = t_i^{\text{inf}}, \qquad (x_{\text{trigger}}, y_{\text{trigger}}) = (x_i, y_i)$$

The campaign is then initiated at the following time t_{vax} and protection develops at t_{prot}, where:

$$t_{\text{vax}} = t_{\text{trigger}} + \tau_{\text{hosp}} + \delta_{\text{logistical}} \text{ and}$$

$$t_{\text{prot}} = t_{\text{vax}} + d_V$$

where d_V is the delay from vaccination to protective immunity.

Step 5: Applying the spatially-targeted vaccination to infections

All individuals within radius r_{vax} of the trigger coordinates are considered eligible to receive the vaccination during the campaign, with this spatial eligibility calculated as $d_j \le r_{vax}$ where

$d_j = \sqrt{(x_j - x_{\text{trigger}})^2 + (y_j - y_{\text{trigger}})^2}$. Additionally, infections can only be averted if they would otherwise occur after the spatial vaccination campaign is initiated, i.e. $t_j^{\text{inf}} \geq t_{\text{vax}}$ and after protection from vaccination has developed i.e. $t_j^{\text{inf}} \geq t_{prot}$. If these criteria are met, the infection is vaccinated with probability c_{cov} . During this vaccination campaign (which occurs only once, in response to the first trigger infection), a number of different outcomes are therefore possible individuals that would otherwise be infected:

1) The individual is successfully vaccinated during the vaccination campaign, protection arises before they would otherwise be infected, and their infection is successfully averted **(Outcome A)**.
2) The individual is successfully vaccinated during the vaccination campaign, protection arises before they would otherwise be infected, but this protection fails to avert their infection **(Outcome B)**.
3) The individual is successfully vaccinated during the vaccination campaign but infected before vaccine-derived protection arises **(Outcome C)**.
4) The individual is infected before the vaccination campaign can be carried out **(Outcome D)**.
5) The individual is not contained within the spatial radius of the vaccination campaign or refuses the vaccine (the latter here is assumed here to be 20% of contacts) **(Outcome E)**.

Below, we define formally the mathematical conditions under which each of these outcomes arises:

**Outcome**	**Verbal description**	**Mathematical condition**
**A** – Infection averted	Vaccinated, protected in time, infection prevented	$d_j \leq R_{\text{camp}}, t_j^{\text{inf}} > t_{\text{prot}}, \text{Bernoulli}(V_I) = 1$
**B** – Vaccinated breakthrough	Vaccinated, protected in time, infection occurs	$d_j \leq R_{\text{camp}}, t_j^{\text{inf}} > t_{\text{prot}}, \text{Bernoulli}(V_I) = 0$
**C** – Vaccinated too late	Vaccinated but infection precedes protection	$d_j \leq R_{\text{camp}}, t_{\text{vax}} \leq t_j^{\text{inf}} \leq t_{\text{prot}}$
**D** – Infection before campaign	Infection occurs before vaccination can start	$t_j^{\text{inf}} < t_{\text{vax}}$
**E** – Outside radius / not vaccinated	Infection occurs outside the vaccination campaign radius **or** refuses the vaccine.	$\left(d_j > R_{\text{camp}}\right) \vee \left(\text{Bernoulli}(c_{\text{cov}}) = 0\right)$

Assumptions of BPSV properties and its impact on reduced transmissibility in breakthrough infections are the same as for the ring-vaccination strategy. Offspring not prevented by vaccination remain in the epidemic tree; and following implementation of the vaccination campaign, the algorithm returns to step (i) to process the next earliest infection (either until 10,000 infections are reached or there are no longer any infections to select).

Box S2: Pseudocode Description of the Spatially-Targeted Vaccination Campaign

```
Input:  N (population size), I0 (seed infections), epidemic_threshold, offspring distribution,
        spatial kernel, natural-history parameters, quarantine parameters
        intervention parameters (vaccination radius Rcamp, coverage c_vax, VE against infection VI,
                                VE against transmission VT, hosp. trigger Hthr, logistical delay δlog,
                                protect delay dV),

Create I0 seed cases at (0,0). Set campaign_triggered ← FALSE, cumulative_hosp ← 0.
while total_cases < check_final_size and S > 0 do
    i ← select earliest unsimulated infection

  # Generate spatially located offspring
    Draw Ni from offspring_distribution(R0).
    If i is breakthrough → keep each child with P = 1 – VT
    Assign infection times via generation time distribution and coordinates via spatial_kernel.

  # Quarantine logic (same as ring model)
    Determine if i isolates; cull offspring ≥ t_iso with efficacy EQ

  # Track hospitalisations and decide if campaign starts
    Draw hospitalisation flag Hi.  cumulative_hosp += Hi
    if !campaign_triggered and cumulative_hosp ≥ Hthr then
        campaign_triggered ← TRUE
        trigger_time  ← t_inf_i + δlog          # vaccination start
        t_prot_start  ← trigger_time + dV
        (x_trig, y_trig) ← coordinates of i
    end if

  # Apply spatial vaccination
    for each surviving offspring j do
        if campaign_triggered
           and distance((xj,yj),(x_trig,y_trig)) ≤ Rcamp
           and t_inf_j ≥ trigger_time then
               Vaccinate with probability c_vax
               if vaccinated and t_inf_j > t_prot_start and Bernoulli(VI)=1
                   discard j                     # infection averted
               else if vaccinated and t_inf_j > t_prot_start
                   mark j as breakthrough        # reduced transmission
               end if
        end if
    end for

    Insert retained offspring into dataframe; update S
end while
Return complete infection-history dataframe
```

1.4 Branching Process Vaccination Parameterisation, Simulations & Analysis

For both strategies, we simulate the proportion of outbreaks contained (defined as a final size of <10,000 infected individuals) and explore the prospects for containment whilst varying:

- **Pathogen Epidemiological Properties:** including the basic reproduction number, generation time distribution, extent of pre-symptomatic transmission, proportion of asymptomatic infections and proportion of infections that are hospitalised.
- **Intrinsic BPSV Properties:** including vaccine efficacy against infection and against onwards transmission (i.e. the degree of reduced transmissibility in breakthrough infections) as well as the delay between vaccination and protection developing.
- **Vaccination Campaign-Related Factors:** including the spatial kernel (namely the size of the vaccination campaign radius relative to the average distance between infections) and surveillance system sensitivity (determining the number of hospitalised cases triggering the spatial vaccination campaign).

In all instances, presented results are based on the proportion of outbreaks successfully contained across 100 stochastic simulations per parameter combination and vaccination strategy considered; and these results are compared to scenarios in which the BPSV is not available (i.e. there is no vaccination). Models were implemented in the programming language R and code required to reproduce the simulations presented in this work is available at https://github.com/mrc-ide/diseaseX_modelling

Table S1: Description of the key parameters varied during branching process analyses of ring and spatially targeted BPSV vaccination. Central value describes the fixed value used during sensitivity analysis of other parameters; range describes the set of parameter values explored during the sensitivity analysis for that particular parameter.

	Central Value	Sensitivity Analysis Range	Description	Notes
**Pathogen Parameters**				
R_0	Varied	1.25 – 2.5	Basic reproduction number, describing the average number of secondary infections generated by an index infection.	
τ_{inf}	α_{Gt} = 12 days "SC1-like"; α_{Gt} = 6.75 days "SC2-like" β_{Gt} = 2 in both case	See Notes on RHS	Specified by the generation time distribution, which describes the distribution of infection times between index infections and secondary infections. Mean of the distribution is given by $\frac{\alpha_{Gt}}{\beta_{Gt}}$	A series of sensitivity analyses were carried out varying T_G for "SC2-like Pathogen" pathogen whilst keeping the incubation period T_I – which in turn affects the proportion of presymptomatic transmission occurring. Varied between 15% and 70% presymptomatic transmission.
τ_{inc}	α_{inc} = 12 days "SC1-like" α_{inc} =2.25 days "SC2-like"	See Notes on RHS	Incubation period distribution, describing the distribution of times between infection and symptom onset in non-asymptomatic individuals. Mean of the distribution is given by $\frac{\alpha_{inc}}{\beta_{inc}}$	Selected to give central values of 0% presymptomatic transmission for "SC1-like" and 34% presymptomatic transmission for "SC2-like".
p_{asymp}	0% for "SC1-Like", 15% for "SC2-Like".	Held constant	Probability that an infection is asymptomatic.	
**BPSV Parameters**				
V_T	35%	30%-90%	BPSV efficacy against onwards transmission i.e. the BPSV-mediated reduction in transmissibility in vaccinated individuals who become infected (breakthrough infections)	Efficacy against infection and efficacy against onwards transmission assumed to covary. Sensitivity analyses of efficacy involve varying these two parameters together.

V_I	35%	30%-90%	BPSV efficacy against infection.	
d_V	Varied	0 days – 28 days	Delay (in days) between BPSV vaccination and protection developing	Time between receiving vaccination and immunological protection developing. We assume here that the BPSV is delivered as a single dose regimen.
**Quarantine Parameters**				
$\tau_{\text{iso,ct}}$ & $\tau_{\text{iso,symp}}$	α_{iso} = 3.34<br>β_{iso} = 0.95	Held constant	Specified by the symptom-to-isolation time distribution, which describes the distribution of times between symptoms developing and isolation occurring. Mean of the distribution is given by $\frac{\alpha_{iso}}{\beta_{iso}}$	Derived from fitting a Gamma distribution to the empirical distribution observed for SARS-CoV-2 and described in Kucharski et al*(3)*.
$p_{iso,ct}$	47%	Held constant	If the index case's *infector* had symptoms, public-health tracing reaches the index (infected by the index case's *infector*) and successfully leads to isolation/quarantine with probability $p_{iso,ct}$.	Calculated based on Kurcharski et al*(3)* – approx. 53% of contacts assumed to be successfully traced, multiplied by an assumed 90% adhering to requests to isolate (to match $p_{iso,symp}$ described below).
$p_{iso,symp}$	90%	Held constant	If the index case itself becomes symptomatic, the index self-isolates with probability $p_{iso,symp}$.	Matching Kurcharski et al*(3)*.
E_Q	Varied	Either 0% (no quarantine/isolation) or 65%.	Quarantine efficacy i.e. the % reduction in transmission that occurs following quarantine and isolation.	Note that this only applies to infections that would otherwise occur after isolation occurs i.e. $t_j^{\text{inf}} > t_{index}^{iso}$
**Vaccination Campaign Parameters**				
$\delta_{\text{logistical}}$	2 days	Held constant	The logistical delay between symptomatic case being identified and vaccination of contacts (in ring vaccination strategy) or individuals residing in affected zone (in spatial vaccination strategy).	
c_{cov}	80%	Held constant	Probability that an individual eligible to be vaccinated is successfully vaccinated (i.e. vaccine is offered and individuals accept the vaccination).	
$k(r)$	Negative binomial with mean of 10 and size parameter of 5.		1-dimensional distance kernel describing the distribution of distances separately the infector and infectee.	A series of sensitivity analyses were carried out varying the spatial vaccination campaign radius (r_{vax}) relative to the mean of this distribution (which was selected arbitrarily).
r_{vax}	25x	1x – 100x	The spatial vaccination campaign radius relative to the mean of $k(r)$.	Expressed as the ratio of the spatial vaccination campaign area relative to the mean of the spatial kernel distribution.

H_{Thresh}	10	1 – 100	The cumulative number of hospitalised infections required to trigger the spatial vaccination campaign.	

2. Supplementary Methods: Dynamical Compartmental Modelling Framework

2.1 Overview of Modelling Framework

We explored the potential utility of the BPSV in vaccination campaigns focussed on rapid mass-vaccination of priority groups following pathogen detection to support disease burden reduction and relaxation of societal restrictions imposed to control transmission (as has been the case with SARS-CoV-2 vaccination campaigns). To do this, we adapted a previously published dynamical model of SARS-CoV-2 transmission used to evaluate and explore the impact of SARS-CoV-2 transmission on COVID-19 mortality during the pandemic *(4)*.

Briefly, the model is an age-stratified SEIRS (susceptible-exposed-infectious-recovered-susceptible) model that explicitly models the progression of COVID-19 disease severity, the transition through various levels of healthcare, and the introduction of vaccination campaigns. A model diagram of transmission with disease and healthcare pathways is given in Figure S12. Complete details of the previous versions of the model are given in *(5–7)* and we focus below on the tranmssion and healthcare pathways and the alterations made to adapt vaccinations to simulate a future, hypothetical SARS-X pandemic and deployment of both a BPSV and (later) a disease-specific vaccine against the pathogen.

2.1.1 Model structure and details

2.1.1.1 Transmission

Transmission between age-groups depends on age-based country-specific contact matrices*(7)*, assuming a specified constant transmission rate per contact. Age groups are 5 yearly up to 80+ years old. Other risk groups or settings (such as healthcare workers or care homes) are not included. The model includes an explicit latent period between an individual becoming infected and subsequently becoming infectious, as well as age-dependent probabilities of hospitalisation and disease severity. The model also captures the loss of infection-derived immunity, assuming this follows an Erlang-2 distribution. Waning is better distributed by an Erlang distribution with a high shape parameter over the exponential distribution created by a compartmental ODE model*(8)*. An Erlang distribution can be simulated by chaining the shape parameters worth of compartments in a row in an ordinary differential equation model. However, simulating more compartments incurs a greater computational cost, so we chose to limit this to 2 compartments, hence using an Erlang-2 distribution to simulate waning. These compartments are shown in Figure S13 and parameters are given in Table S2.

2.1.1.2 Healthcare and Disease

In terms of modelling disease progression and differential disease severity, the modelling framework explicitly includes elements of the clinical pathway differentiating individuals by disease severity within clinical settings (e.g. those requiring a basic hospital bed and limited oxygen consumption, as well as those requiring a protracted ICU stay and mechanical ventilation). The model assumes that all COVID-19 related deaths occur in those requiring hospitalisation. The values for these parameters are given in Table S2. This is depicted in Figure S12.

2.1.1.3 Vaccination

The model's vaccination pathway incorporates both a delay in the development of protection following vaccination, as well as the possibility of waning of vaccine protection. Both of these are assumed to follow an Erlang distribution with a shape of two and a mean duration controlled by a model parameter that can be altered to reflect different assumptions around waning. Susceptible, latent, and recovered individuals can be vaccinated. Stepped vaccination strategies prioritising different age groups can be simulated. Because latent individuals can be vaccinated, it is possible for latent individuals to develop vaccine derived protection before realising their infection. However, due to short duration of a latent infection in comparison to the delay in developing vaccine-derived protection, the size of this group relative to susceptible or recovered individuals is small and the effect of this is minor.

2.1.2 Modelling BPSV and Disease-Specific Vaccine Distribution

Following activation of BPSV stockpiles, eligible individuals (here considered to be all those aged 60+) are vaccinated with the BPSV at a constant rate and to a level of coverage determined by the size of the BPSV stockpile relative to the size of the eligible population. We assume here that the BPSV is delivered as a single dose regimen but note that the model is flexibly able to accommodate a wide variety of delays in the development of protection through the delayed protection mechanisms described in the paragraph above. In all scenarios considered, the BPSV is only delivered to the 60+ population – individuals below this age do not receive it. Following introduction

of the disease-specific vaccine, elderly individuals at the greatest risk (again, those aged 60+) are prioritised to receive the disease-specific vaccine – and both elderly individuals who received the BPSV and those who did not (e.g. because the size of the stockpile precluded it) are vaccinated. Following complete vaccination of the elderly high-risk groups with the disease-specific vaccine, the disease specific vaccine is then rolled out and distributed to all other age-groups > 15 years.

2.1.3. Modelling BPSV and Disease-Specific Vaccination Effectiveness

We model two distinct forms of vaccine efficacy: efficacy against infection and efficacy against severe disease (reducing risk of hospitalisation and death) in breakthrough infections (i.e. individuals who were vaccinated but where vaccination failed to prevent the infection occurring). We make the assumption that protection is partial, and that vaccine efficacy is the same for all individuals considered. For those protected by both vaccine-derived and infection-derived immunity, we assume that the most protective effect is dominant – though note given the timeframes over which we simulate (typically no longer than 18 months), we expect minimal waning of immunity to have occurred.

2.2 Hypothetical SARS-X Pandemic Scenario Construction & Baseline Parameterisation

We use this modelling framework to explore the potential impact of BPSV availability on disease burden during a future hypothetical SARS-X pandemic. In this hypothetical scenario, the BPSV has been manufactured and stockpiled ahead of the pandemic, enabling rapid deployment following pathogen detection (which is the trigger for initiation of the BPSV vaccination campaign). We also explicitly model a pathogen-specific vaccine, which we assume to have a more favourable efficacy profile than the BPSV but that can only be developed following detection of the novel pathogen and sequencing of its genome. It is therefore only available after a significant delay. Our baseline scenario assumes the BPSV has 75% efficacy against severe disease and 35% efficacy against infection whilst a future disease-specific vaccine is assumed to have vaccine efficacy of 95% against severe disease and 55% against infection. In both instances, we assume there is minimal waning of vaccine-derived immunity over the timescale of the simulation period considered and we assume that it takes 7 days for development of immunological protection to occur following receipt of the vaccine. For the disease-specific vaccine we explore a development timeline of either 250 or 100 days (reflecting recent estimates from CEPI around realistic and ambitious vaccine development timelines *(9)*). In our simulations, pathogen spillover is followed by a period of undetected circulation in the community. Hospitalisations due to the infection lead to pathogen detection and identification. We assumed detection occurred when daily incidence of hospitalised individuals reached 5 hospitalisations per day. The time for this to occur following spillover was calculated using the stochastic branching-process based framework described above.

Following pathogen identification, development of the pathogen-specific vaccine starts, and after an assumed delay of 7 days (reflecting delays around decision making and activation of stockpiles), mass vaccination of the elderly population (here assumed to be those aged 60+) with the BPSV begins. The size of the BPSV stockpile is assumed sufficient to vaccinate 80% of the elderly population, with health systems capabilities able to vaccinate the population at a rate of 3.5% of the population per week, leading to completion of BPSV vaccination campaign within 3 weeks. All age-groups except those under 15 are eligible to receive the disease-specific vaccine, with rollout of this vaccine (sufficient to achieve a coverage of 80% of the population) occurring in the oldest age-groups first. We assume an R0 of 2.5 and an average generation time of 6.7 days, in-keeping with estimates derived for the original Wuhan-1 strain, as well as a severity profile and age-specific IFR similar to that of SARS-CoV-2 *(10)*, adjusted to give an overall population-level IFR of 1%. We assume a demographic population age-structure matching the age-distribution of the World Bank Upper Middle-Income Country with the median age, and for the purposes of our scenarios assume no healthcare constraints that limit the ability of hospitalised individuals to access adequate medical care. We note that relaxing this assumption and imposing limited healthcare availability (and associated excess mortality risk arising from insufficient medical care) would only serve to increase our estimates of BPSV impact, and that previous analyses exploring the impact of COVID-19 vaccination on mortality have shown that direct protection was the main driver of deaths averted, with very few averted by the reduction in healthcare capacity required *(4)*.

We explore 9 different scenarios varying the duration, stringency and timing of non-pharmaceutical interventions imposed in response to identification of the novel pathogen (shown in **Figure 3B**). Three stringency levels are considered: i) no NPIs (and so R is equal to R0); ii) a minimal and limited set of NPIs reducing transmission by 25%; and iii) a stringent set of NPIs sufficient to reduce Rt to 0.9. Whilst **Figure 3** considers 9 scenarios spanning a wide range of possible NPI responses, we use three central NPI scenarios for the rest of the analyses presented here. These are 1) "minimal NPI scenario" which assumes a short imposition of limited NPIs between pathogen identification and completion of the BPSV campaign; a 2) "moderate NPI scenario" involving imposition of the limited NPIs in response to pathogen identification. These then last in full until the end of BPSV campaign, whereafter they are gradually released and relaxed, and lift completely upon completion of the disease-specific vaccination campaign; and a 3) "stringent NPI scenario"

which sees stringent NPIs implemented until the BPSV campaign is complete, followed by imposition of more limited NPIs which gradually lift until the disease-specific vaccination campaign is complete. We additionally carried out a series of sensitivity analyses varying a number of the model parameters described above. In all cases, we varied model parameters in a univariate manner whilst keeping all other parameter estimates identical to the baseline parameters described above. A detailed description of the exact parameters varied, and the parameter ranges used are described in **Table 1** and in the **Supplementary Table 2**.

For all scenarios, we calculated the deaths averted due to BPSV vaccination by subtracting the estimated SARS-X deaths from the simulation with both BPSV and disease-specific vaccines from the estimated number of SARS-X deaths under a scenario where only the disease-specific vaccine is available and report the number of deaths averted per 1,000 population. To calculate the NPI index featured in **Figure 3 of the main text** we construct a composite measure that considers both the duration and stringency of NPIs imposed in response to the hypothetical pandemic. This composite measure was calculated by first calculating the relative stringency of each set of NPIs, defined as the % reduction in the R0 by the NPIs (with a higher % reduction reflecting more stringent and costly NPIs). We then multiplied this stringency by the number of days spent under those NPIs to construct an index considering both stringency and duration of NPIs.

2.3 Retrospective Evaluation of Potential Impact During SARS-CoV-2 Pandemic

Using previously published model fits calibrated within a Bayesian framework to excess mortality data (known to be a more complete measure of pandemic mortality, especially in LMIC settings with less robust vital registration) *(5)*, we explored the potential impact that a stockpiled BPSV could have had on COVID-19 mortality in the first year of the pandemic. We used the resulting model fits to estimate the time-varying reproductive number, Rt, and its associated uncertainty by sampling 100 draws from the estimated posterior distribution of Rt from these previous fits. To estimate the impact of BPSV, we simulated a counterfactual scenario for each sampled Rt trajectory in which BPSV were introduced following globally reported COVID-19 deaths reached a certain threshold (varied between 1 and 1000 cumulative global deaths), under the assumption that all countries have access to a BPSV stockpile sufficient to vaccinate 60% of their eligible elderly population (here assumed to be those aged 60+) at a rate representing the average COVID-19 vaccination rate for each World Bank Income Strata based on data from Our World In Data *(11)*; and under the strong assumption that availability of the BPSV would not have altered the NPIs imposed in response to SARS-CoV-2 (and hence alterations to Rt due to NPIs would be the same across both scenarios). We then calculated the deaths averted as a result of BPSV vaccination by subtracting the estimated COVID-19 deaths from the simulation with BPSV vaccines included from the estimated COVID-19 deaths from the simulation with only the real-world vaccination campaign included and reported the median deaths averted per 1,000 population.

Table S2: Description of model parameters varied in dynamical compartmental modelling of mass-vaccination of high-risk populations with BPSV. Central value describes the fixed value used during sensitivity analysis of other parameters; range describes the set of parameter values explored during the sensitivity analysis for that particular parameter. Parameter estimates were selected to replicate the approximate epidemiological properties of SARS-CoV-2, and complete set of model parameters used can be found https://github.com/mrc-ide/diseaseX_modelling.

	**Central Value**	**Sensitivity Analysis Range**	**Notes**
**Epidemiological & Pathogen Parameters**			
Basic reproduction number (R0)	2.5	1.5 – 3.5	
NPI Scenarios	3 NPI scenarios	9 NPI scenarios considered, ranging in stringency and duration **(Fig 3B & Fig 3C)**	In all cases, minimal mandate controls are assumed to reduce transmissibility by 25%, and stringent measures assumed to bring Rt to 0.9.<br>3 central NPI scenarios are:<br>**Minimal NPIs:** 25% reduction in Rt during BPSV campaign. No NPIs thereafter.<br>**Moderate NPIs:** 25% reduction in Rt during BPSV campaign. Slow cessation of NPIs that finishes when disease-specific vaccination campaign completes.<br>**Stringent NPIs:** Reduction in Rt to 0.9 during BPSV campaign. Slow cessation of NPIs that finishes when disease-specific vaccination campaign completes.

Surveillance System Sensitivity	Daily incidence of 5 hospitalisations to trigger pathogen detection	Daily incidence of 1-50 hospitalisations to trigger pathogen detection	
**Target Product Characteristics & Vaccine Development-Related Parameters**			
BPSV disease efficacy	75%	10-100% **(Fig 5A)**	BPSV efficacy against severe disease in breakthrough infections (i.e. where the BPSV fails to prevent the infection). Central value for BPSV assumed lower than disease-specific vaccine efficacy against severe-disease (95%)
BPSV efficacy against infection	35%	10% - 100% **(Fig 5B)**	BPSV efficacy against being infected. Central value for BPSV assumed lower than disease-specific vaccine efficacy against infection (55%)
Duration of BPSV-induced immunity	365 days	30-180 days **(Fig 5C)**	365 days selected to reflect the assumption of minimal waning over the period between BPSV vaccination and introduction of the disease-specific vaccine 250 days later. Central value assumes minimal waning over the 100–250 day period between BPSV vaccination starting and the disease-specific alternative vaccine becoming available.
**Operational, Vaccination Campaign & Access-Related Parameters**			
Time to develop the disease-specific vaccine	250 days	100 – 365 days **(Fig 3C and Fig 5D)**	Time to develop the disease specific vaccine following pathogen identification and genomic sequencing. 100 days and 250 days selected as central scenarios **(in Fig 3C)** and the full range explored in a sensitivity analyses in **Fig 5D.** Selected based on CEPI report of optimistic and realistic scenarios for improvements to vaccine development timelines *(12)*
Size of BPSV stockpile	80%	10-80% **(Fig 5A)**	Describes the size of the BPSV stockpile and the associated level of BPSV coverage able to be achieved in the 60+ year old population eligible to receive the vaccine.
Speed of BPSV vaccination campaign	3.5% of population per week	0.5% - 4.5% of population per week **(Fig 5B)**	Central scenario corresponds to taking 4 weeks to vaccine entire 60+ years age-group. Range corresponds to taking 20 – 170 days. Central value based on estimates derived from COVID-19 vaccination data from Our World In Data *(13)*
Delay to Accessing Disease-Specific Vaccine	0 days	5 days – 180 days	Assume local vaccine manufacturing capabilities in place for central value. Sensitivity analysis range derived from COVID-19 vaccination data from Our World In Data *(13)*
**Disease Progression & Clinical Parameters**			
Average Incubation Period	4.6 days		Estimated to be 5.1 days*(14, 15)*. The last 0.5 days are included in the duration of infectiousness to capture pre-symptomatic infectivity
Average Duration of Infectiousness	Mild: 2.1 days		Mild: As majority of cases are mild, this is derived from a generation time of 6.75 days*(16)*

	Hospitalised: 4.5 days		Hospitalised: Assumed time from symptoms to hospitalisation of 4 days based on UK data *(17–19)*
Probability of requiring Hospitalisation, given Infection	Age-dependent, see Figure 2 in reference		Sourced from *(20)*
Probability of requiring Ventilation, given Hospitalisation	Age-dependent, see Figure 2 in reference		Sourced from *(20)*
Probability of death if untreated	Require Hospitalisation: 60%<br><br>Requires Ventilation: 95%		Assumption based on expert clinical opinion
Average Duration of Hospitalisation	Survive: 9 days<br><br>Die: 9 days		Median value from four studies *(21–24)*
Average Duration of Hospitalisation, if given Ventilation	Survive: 14.8 days<br><br>Die: 11.1 days		Derived from a mean duration of ICU stay of 13.3 and ratio of duration of stay by survival *(25)*
Average Duration of step-down ICU if given Ventilation	3 days		Assumption based on unpublished UK data

Dynamical Compartmental Modelling Framework Equations

1 Age groups and Vaccination Statuses

The model includes 17 age groups (indexed by a) and 8 vaccination statuses (indexed by v), where:

- $v = 1$: Unvaccinated
- $v = 2$: First dose received
- $v = 3$: Second dose received
- $v = 4, 5$: Waned immunity states
- $v = 6$: First booster received
- $v = 7, 8$: Further waned booster states

2 Model Compartments

The model consists of the following compartments for each age group a and vaccination status v:

- $S_{a,v}$: Susceptible individuals
- $E1_{a,v}$, $E2_{a,v}$: Exposed individuals (latent infection stages)
- $I^{\text{Mild}}_{a,v}$: Mild infections (non-hospitalized)
- $I1_{a,v}$, $I2_{a,v}$: Severe infections (pre-hospitalization)
- $R1_{a,v}$, $R2_{a,v}$: Recovered individuals
- $D_{a,v}$: Deaths

Hospital and ICU compartments (detailed in Section 4):

- Hospitalised compartments: $H^{\text{Hosp,Get,Live}}_{a,v}$, $H^{\text{Hosp,Get,Die}}_{a,v}$, $H^{\text{Hosp,NotGet,Live}}_{a,v}$, $H^{\text{Hosp,NotGet,Die}}_{a,v}$

- ICU compartments: $H_{a,v}^{\text{ICU,Get,Live}}$, $H_{a,v}^{\text{ICU,Get,Die}}$, $H_{a,v}^{\text{ICU,NotGet,Live}}$, $H_{a,v}^{\text{ICU,NotGet,Die}}$
- Recovery (from ICU) compartments: $H1_{a,v}^{\text{Rec}}$, $H2_{a,v}^{\text{Rec}}$

Please note that compartment values vary over time, but this notation is not included for brevity, i.e. $S_{a,v}$ is truely $S_{a,v}(t)$.

3 Core Epidemiological Dynamics

3.1 Susceptible Compartment

$$\frac{dS_{a,1}}{dt} = \gamma_R(t)R2_{a,1} - \lambda_a\xi_1^{\text{inf}}(t)S_{a,1} - \gamma_1^{\text{vac}}(t)S_{a,1} + V_{a,1}^S$$

$$\frac{dS_{a,v}}{dt} = \gamma_R(t)R2_{a,v} - \lambda_a\xi_v^{\text{inf}}(t)S_{a,v} - \gamma_v^{\text{vac}}(t)S_{a,v} + V_{a,v}^S + \gamma_{v-1}^{\text{vac}}(t)S_{a,v-1}$$

for $v = 2, \ldots, 8$
where:

- λ_a is the force of infection for age group a
- $\xi_v^{\text{inf}}(t)$ is the vaccine efficacy against infection for vaccination status v
- $\gamma_v^{\text{vac}}(t)$ is the rate of vaccine waning for status v
- $\gamma_R(t)$ is the rate of loss of natural immunity
- $V_{a,v}^S$ represents vaccination transitions

3.2 Exposed Compartments

$$\frac{dE1_{a,1}}{dt} = \lambda_a\xi_1^{\text{inf}}(t)S_{a,1} - \gamma_E E1_{a,1} - \gamma_1^{\text{vac}}(t)E1_{a,1} + V_{a,1}^{E1}$$

$$\frac{dE1_{a,v}}{dt} = \lambda_a\xi_v^{\text{inf}}(t)S_{a,v} - \gamma_E E1_{a,v} - \gamma_v^{\text{vac}}(t)E1_{a,v} + V_{a,v}^{E1} + \gamma_{v-1}^{\text{vac}}(t)E1_{a,v-1}$$

for $v = 2, \ldots, 8$

$$\frac{dE2_{a,1}}{dt} = \gamma_E E1_{a,1} - \gamma_E E2_{a,1} - \gamma_1^{\text{vac}}(t)E2_{a,1} + V_{a,1}^{E2}$$

$$\frac{dE2_{a,v}}{dt} = \gamma_E E1_{a,v} - \gamma_E E2_{a,v} - \gamma_v^{\text{vac}}(t)E2_{a,v} + V_{a,v}^{E2} + \gamma_{v-1}^{\text{vac}}(t)E2_{a,v-1}$$

for $v = 2, \ldots, 8$
where γ_E is the rate of progression through latent infection.

3.3 Mild Infections

$$\frac{dI^{\text{Mild}}_{a,1}}{dt} = \gamma_E E2_{a,1}(1 - p^{\text{hosp}}_{a,1}(t)) - \gamma^{\text{Mild}} I^{\text{Mild}}_{a,1} - \gamma^{\text{vac}}_1(t) I^{\text{Mild}}_{a,1}$$

$$\frac{dI^{\text{Mild}}_{a,v}}{dt} = \gamma_E E2_{a,v}(1 - p^{\text{hosp}}_{a,v}(t)) - \gamma^{\text{Mild}} I^{\text{Mild}}_{a,v} - \gamma^{\text{vac}}_v(t) I^{\text{Mild}}_{a,v} + \gamma^{\text{vac}}_{v-1}(t) I^{\text{Mild}}_{a,v-1}$$

for $v = 2, \ldots, 8$
where:

- $p^{\text{hosp}}_{a,v}(t) = p^{\text{hosp}}_a \cdot \xi^{\text{dis}}_v(t) \cdot m^{\text{hosp}}(t)$ is the time-varying hospitalization probability
- γ^{Mild} is the recovery rate from mild infection

3.4 Severe Infections (Pre-hospitalization)

$$\frac{dI1_{a,1}}{dt} = \gamma_E E2_{a,1} p^{\text{hosp}}_{a,1}(t) - \gamma^{\text{Case}} I1_{a,1} - \gamma^{\text{vac}}_1(t) I1_{a,1}$$

$$\frac{dI1_{a,v}}{dt} = \gamma_E E2_{a,v} p^{\text{hosp}}_{a,v}(t) - \gamma^{\text{Case}} I1_{a,v} - \gamma^{\text{vac}}_v(t) I1_{a,v} + \gamma^{\text{vac}}_{v-1}(t) I1_{a,v-1}$$

$$\frac{dI2_{a,1}}{dt} = \gamma^{\text{Case}} I1_{a,1} - \gamma^{\text{Case}} I2_{a,1} - \gamma^{\text{vac}}_1(t) I2_{a,1}$$

$$\frac{dI2_{a,v}}{dt} = \gamma^{\text{Case}} I1_{a,v} - \gamma^{\text{Case}} I2_{a,v} - \gamma^{\text{vac}}_v(t) I2_{a,v} + \gamma^{\text{vac}}_{v-1}(t) I2_{a,v-1}$$

for $v = 2, \ldots, 8$

3.5 Recovered Compartments

$$\begin{aligned}\frac{dR1_{a,1}}{dt} = {} & \gamma^{\text{rec}} H2^{\text{Rec}}_{a,1} + \gamma^{\text{Mild}} I^{\text{Mild}}_{a,1} + \gamma^{\text{Hosp,surv}}(t) H2^{\text{Hosp,Get,Live}}_{a,1} \\ & + \gamma^{\text{Hosp,surv}} H2^{\text{Hosp,NotGet,Live}}_{a,1} + \gamma^{\text{ICU,surv}} H2^{\text{ICU,NotGet,Live}}_{a,1} \\ & - \gamma_R(t) R1_{a,1} - \gamma^{\text{vac}}_1(t) R1_{a,1} + V^{R1}_{a,1}\end{aligned}$$

$$\begin{aligned}\frac{dR1_{a,v}}{dt} = {} & \gamma^{\text{rec}} H2^{\text{Rec}}_{a,v} + \gamma^{\text{Mild}} I^{\text{Mild}}_{a,v} + \gamma^{\text{Hosp,surv}}(t) H2^{\text{Hosp,Get,Live}}_{a,v} \\ & + \gamma^{\text{Hosp,surv}} H2^{\text{Hosp,NotGet,Live}}_{a,v} + \gamma^{\text{ICU,surv}} H2^{\text{ICU,NotGet,Live}}_{a,v} \\ & - \gamma_R(t) R1_{a,v} - \gamma^{\text{vac}}_v(t) R1_{a,v} + V^{R1}_{a,v} + \gamma^{\text{vac}}_{v-1}(t) R1_{a,v-1}\end{aligned}$$

for $v = 2, \ldots, 8$

$$\frac{dR2_{a,1}}{dt} = \gamma_R(t)R1_{a,1} - \gamma_R(t)R2_{a,1} - \gamma_1^{\text{vac}}(t)R2_{a,1} + V_{a,1}^{R2}$$

$$\frac{dR2_{a,v}}{dt} = \gamma_R(t)R1_{a,v} - \gamma_R(t)R2_{a,v} - \gamma_v^{\text{vac}}(t)R2_{a,v} + V_{a,v}^{R2} + \gamma_{v-1}^{\text{vac}}(t)R2_{a,v-1}$$

for $v = 2, \ldots, 8$

4 Hospital and ICU Dynamics

For individuals who do not require ICU, get treatment and survive:

$$\begin{aligned}\frac{dH1_{a,1}^{\text{Hosp,Get,Live}}}{dt} &= \gamma^{\text{Case}}I2_{a,1}(1 - p_a^{\text{sev}}(t))p^{\text{hosp}}(1 - p_a^{\text{death,Hosp,treat}}) \\ &\quad - \gamma^{\text{Hosp,surv}}(t)H1_{a,1}^{\text{Hosp,Get,Live}} - \gamma_1^{\text{vac}}(t)H1_{a,1}^{\text{Hosp,Get,Live}}\end{aligned}$$

$$\begin{aligned}\frac{dH1_{a,v}^{\text{Hosp,Get,Live}}}{dt} &= \gamma^{\text{Case}}I2_{a,v}(1 - p_a^{\text{sev}}(t))p^{\text{hosp}}(1 - p_a^{\text{death,Hosp,treat}}) \\ &\quad - \gamma^{\text{Hosp,surv}}(t)H1_{a,v}^{\text{Hosp,Get,Live}} - \gamma_v^{\text{vac}}(t)H1_{a,v}^{\text{Hosp,Get,Live}} \\ &\quad + \gamma_{v-1}^{\text{vac}}(t)H1_{a,v-1}^{\text{Hosp,Get,Live}}\end{aligned}$$

for $v = 2, \ldots, 8$

$$\frac{dH2_{a,1}^{\text{Hosp,Get,Live}}}{dt} = \gamma^{\text{Hosp,surv}}(t)H1_{a,1}^{\text{Hosp,Get,Live}} - \gamma^{\text{Hosp,surv}}(t)H2_{a,1}^{\text{Hosp,Get,Live}} - \gamma_1^{\text{vac}}(t)H2_{a,1}^{\text{Hosp,Get,Live}}$$

$$\begin{aligned}\frac{dH2_{a,v}^{\text{Hosp,Get,Live}}}{dt} &= \gamma^{\text{Hosp,surv}}(t)H1_{a,v}^{\text{Hosp,Get,Live}} - \gamma^{\text{Hosp,surv}}(t)H2_{a,v}^{\text{Hosp,Get,Live}} \\ &\quad - \gamma_v^{\text{vac}}(t)H2_{a,v}^{\text{Hosp,Get,Live}} + \gamma_{v-1}^{\text{vac}}(t)H2_{a,v-1}^{\text{Hosp,Get,Live}}\end{aligned}$$

for $v = 2, \ldots, 8$

Similar equations exist for:

- $H1_{a,v}^{\text{Hosp,Get,Die}}$, $H2_{a,v}^{\text{Hosp,Get,Die}}$: Get treatment, die
- $H1_{a,v}^{\text{Hosp,NotGet,Live}}$, $H2_{a,v}^{\text{Hosp,NotGet,Live}}$: Don't get treatment, survive
- $H1_{a,v}^{\text{Hosp,NotGet,Die}}$, $H2_{a,v}^{\text{Hosp,NotGet,Die}}$: Don't get treatment, die

4.1 Mechanical Ventilation Compartments

For individuals who get mechanical ventilation and survive:

$$\begin{aligned}\frac{dH1_{a,1}^{\text{ICU,Get,Live}}}{dt} &= \gamma^{\text{Case}} I2_{a,1} p_a^{\text{sev}}(t) p^{\text{ICU}}(1 - p_a^{\text{death,ICU,treat}}) \\ &- \gamma^{\text{ICU,surv}}(t) H1_{a,1}^{\text{ICU,Get,Live}} - \gamma_1^{\text{vac}}(t) H1_{a,1}^{\text{ICU,Get,Live}}\end{aligned}$$

$$\begin{aligned}\frac{dH1_{a,v}^{\text{ICU,Get,Live}}}{dt} &= \gamma^{\text{Case}} I2_{a,v} p_a^{\text{sev}}(t) p^{\text{ICU}}(1 - p_a^{\text{death,ICU,treat}}) \\ &- \gamma^{\text{ICU,surv}}(t) H1_{a,v}^{\text{ICU,Get,Live}} - \gamma_v^{\text{vac}}(t) H1_{a,v}^{\text{ICU,Get,Live}} \\ &+ \gamma_{v-1}^{\text{vac}}(t) H1_{a,v-1}^{\text{ICU,Get,Live}}\end{aligned}$$

for $v = 2, \ldots, 8$

Similar structures exist for all mechanical ventilation compartments.

4.2 Recovery from ICU

$$\frac{dH1_{a,1}^{\text{Rec}}}{dt} = \gamma^{\text{ICU,surv}}(t) H2_{a,1}^{\text{ICU,Get,Live}} - \gamma^{\text{rec}} H1_{a,1}^{\text{Rec}} - \gamma_1^{\text{vac}}(t) H1_{a,1}^{\text{Rec}}$$

$$\begin{aligned}\frac{dH1_{a,v}^{\text{Rec}}}{dt} &= \gamma^{\text{ICU,surv}}(t) H2_{a,v}^{\text{ICU,Get,Live}} - \gamma^{\text{rec}} H1_{a,v}^{\text{Rec}} \\ &- \gamma_v^{\text{vac}}(t) H1_{a,v}^{\text{Rec}} + \gamma_{v-1}^{\text{vac}}(t) H1_{a,v-1}^{\text{Rec}}\end{aligned}$$

for $v = 2, \ldots, 8$

$$\frac{dH2_{a,1}^{\text{Rec}}}{dt} = \gamma^{\text{rec}} H1_{a,1}^{\text{Rec}} - \gamma^{\text{rec}} H2_{a,1}^{\text{Rec}} - \gamma_1^{\text{vac}}(t) H2_{a,1}^{\text{Rec}}$$

$$\begin{aligned}\frac{dH2_{a,v}^{\text{Rec}}}{dt} &= \gamma^{\text{rec}} H1_{a,v}^{\text{Rec}} - \gamma^{\text{rec}} H2_{a,v}^{\text{Rec}} \\ &- \gamma_v^{\text{vac}}(t) H2_{a,v}^{\text{Rec}} + \gamma_{v-1}^{\text{vac}}(t) H2_{a,v-1}^{\text{Rec}}\end{aligned}$$

for $v = 2, \ldots, 8$

4.3 Deaths

$$\begin{aligned}\frac{dD_{a,v}}{dt} &= \gamma^{\text{Hosp,die}}(t) H2_{a,v}^{\text{Hosp,Get,Die}} + \gamma^{\text{Hosp,die}} H2_{a,v}^{\text{Hosp,NotGet,Die}} \\ &+ \gamma^{\text{ICU,die}}(t) H2_{a,v}^{\text{ICU,Get,Die}} + \gamma^{\text{ICU,die}} H2_{a,v}^{\text{ICU,NotGet,Die}}\end{aligned}$$

5 Vaccination Dynamics

5.1 Vaccination Transitions

The vaccination transitions $V_{a,v}^X$ for compartment X are defined as:

$$
\begin{aligned}
V_{a,1}^X &= -r_a^{\text{prim},1} X_{a,1} \\
V_{a,2}^X &= r_a^{\text{prim},1} X_{a,1} - r^{\text{prim},2} X_{a,2} \\
V_{a,3}^X &= r^{\text{prim},2} X_{a,2} - r^{\text{boost},1} X_{a,3} \\
V_{a,4}^X &= -r^{\text{boost},1} X_{a,4} \\
V_{a,5}^X &= -r^{\text{boost},1} X_{a,5} \\
V_{a,6}^X &= r^{\text{boost},1} \sum_{k=3}^{5} X_{a,k} + r_a^{\text{boost},2} \sum_{k=7}^{8} X_{a,k} \\
V_{a,7}^X &= -r_a^{\text{boost},2} X_{a,7} \\
V_{a,8}^X &= -r_a^{\text{boost},2} X_{a,8}
\end{aligned}
$$

where:

- $r_a^{\text{prim},1}$ is the first dose rate for age group a
- $r^{\text{prim},2}$ is the second dose rate
- $r^{\text{boost},1}$ is the first booster rate
- $r_a^{\text{boost},2}$ is the second booster rate for age group a

6 Force of Infection

The force of infection for age group a is:

$$\lambda_a = \beta(t) \sum_{k=1}^{17} M_{a,k} \rho_k \sum_{v=1}^{8} \left(I_{k,v}^{\text{Mild}} + I1_{k,v} + I2_{k,v} \right) \psi_v$$

where:

- $\beta(t)$ is the time-varying transmission rate
- $M_{a,k}$ is the contact matrix between age groups a and k
- ρ_k is the relative infectiousness of age group k
- ψ_v is the relative infectiousness of vaccination status v

7 Time-Varying Parameters

Several parameters in the model are time-varying and implemented using interpolation:

- $\beta(t)$: Transmission rate
- $\gamma_R(t)$: Rate of immunity waning
- $\xi_v^{\text{inf}}(t)$: Vaccine efficacy against infection
- $\xi_v^{\text{dis}}(t)$: Vaccine efficacy against disease
- $\gamma_v^{\text{vac}}(t)$: Vaccine waning rates
- $\gamma^{\text{Hosp,surv}}(t)$, $\gamma^{\text{Hosp,die}}(t)$: (non-ICU) Treatment progression rates
- $\gamma^{\text{ICU,surv}}(t)$, $\gamma^{\text{ICU,die}}(t)$: ICU progression rates
- $m^{\text{hosp}}(t)$: Hospitalization probability multiplier
- $m^{\text{sev}}(t)$: Severity probability multiplier

8 Parameter Definitions

Parameter	Description
γ_E	Rate of progression through latent infection
γ^{Mild}	Recovery rate from mild infection
γ^{Case}	Rate of progression from severe infection
γ^{rec}	Rate of progression through post-ICU recovery
p_a^{hosp}	Probability of hospitalization by age group
p_a^{sev}	Probability of severe disease (requiring ICU treatment) by age group
$p_a^{\text{death,Hosp,treat}}$	Death probability with (non-ICU) treatment
$p_a^{\text{death,Hosp,notreat}}$	Death probability without ICU treatment
$p_a^{\text{death,ICU,treat}}$	Death probability with mechanical ventilation
$p_a^{\text{death,ICU,notreat}}$	Death probability without mechanical ventilation

Table 1: Key model parameters

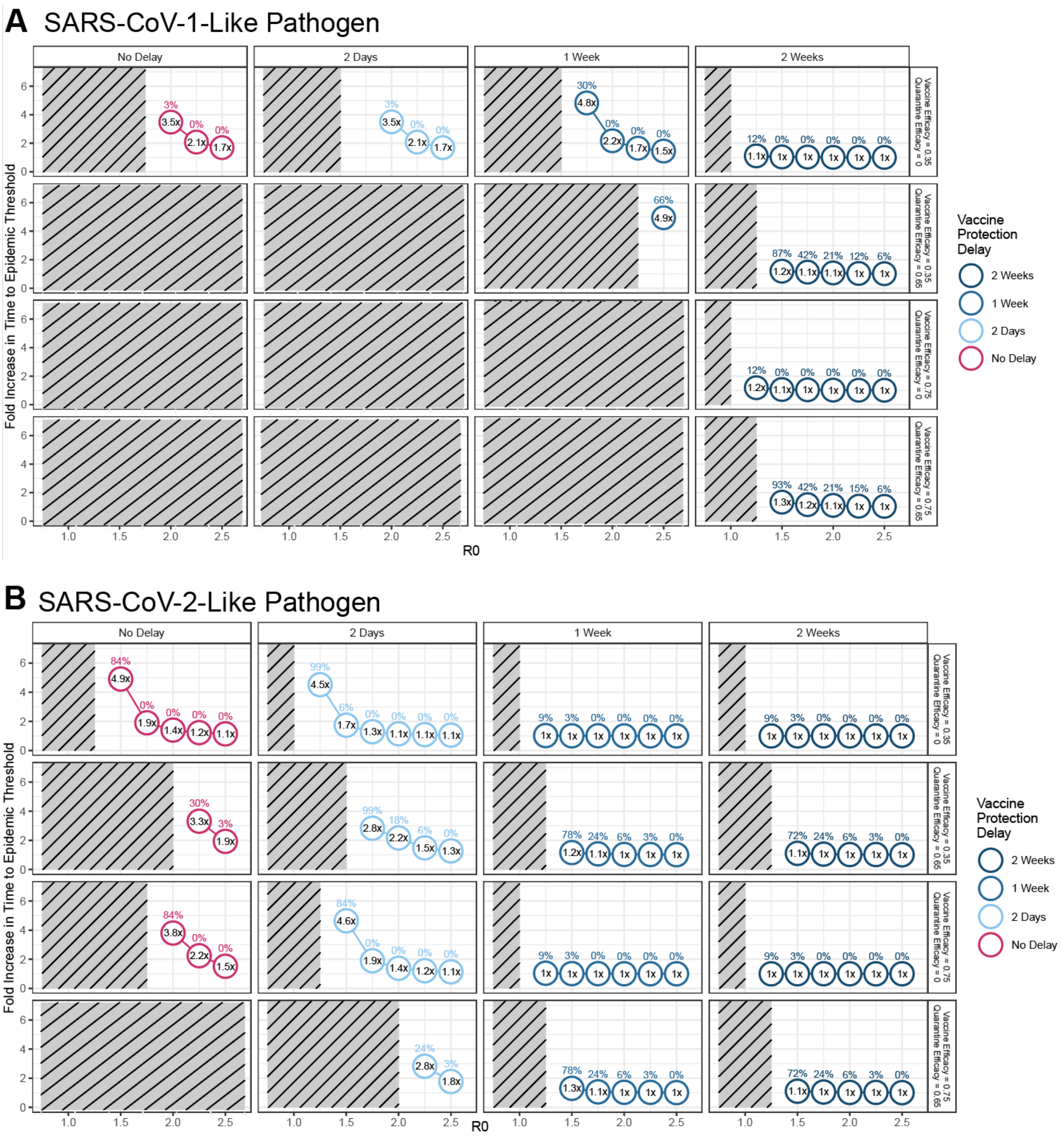

Figure S1: Time to reach the epidemic threshold in uncontrolled epidemics across different ring-vaccination scenarios. (A) Time to reach the epidemic threshold (10,000 infections) for the "SARS-CoV-1-Like" pathogen archetype relative to a no vaccination scenario. Facet columns (and colour) indicate the assumed vaccine protection delay; facet rows the combination of vaccine efficacy against infection (either 35% or 75%) and quarantine/isolation effectiveness (either no quarantine or 65% efficacy). Black text inside each point denotes the fold increase in time to reach 10,000 infections relative to a no vaccination scenario; coloured text above each point indicates the % of outbreaks that are controlled successfully i.e. 0% means no outbreaks were successfully controlled (limited to <10,000 infections). Greyed out area with black lines indicates scenarios where 100% of outbreaks were successfully controlled. Results are based on 100 stochastic simulations, with the mean fold time to increase for unsuccessfully controlled outbreaks plotted inside the circle. **(B)** As for **(A)**, but for the "SARS-CoV-2-Like" pathogen archetype.

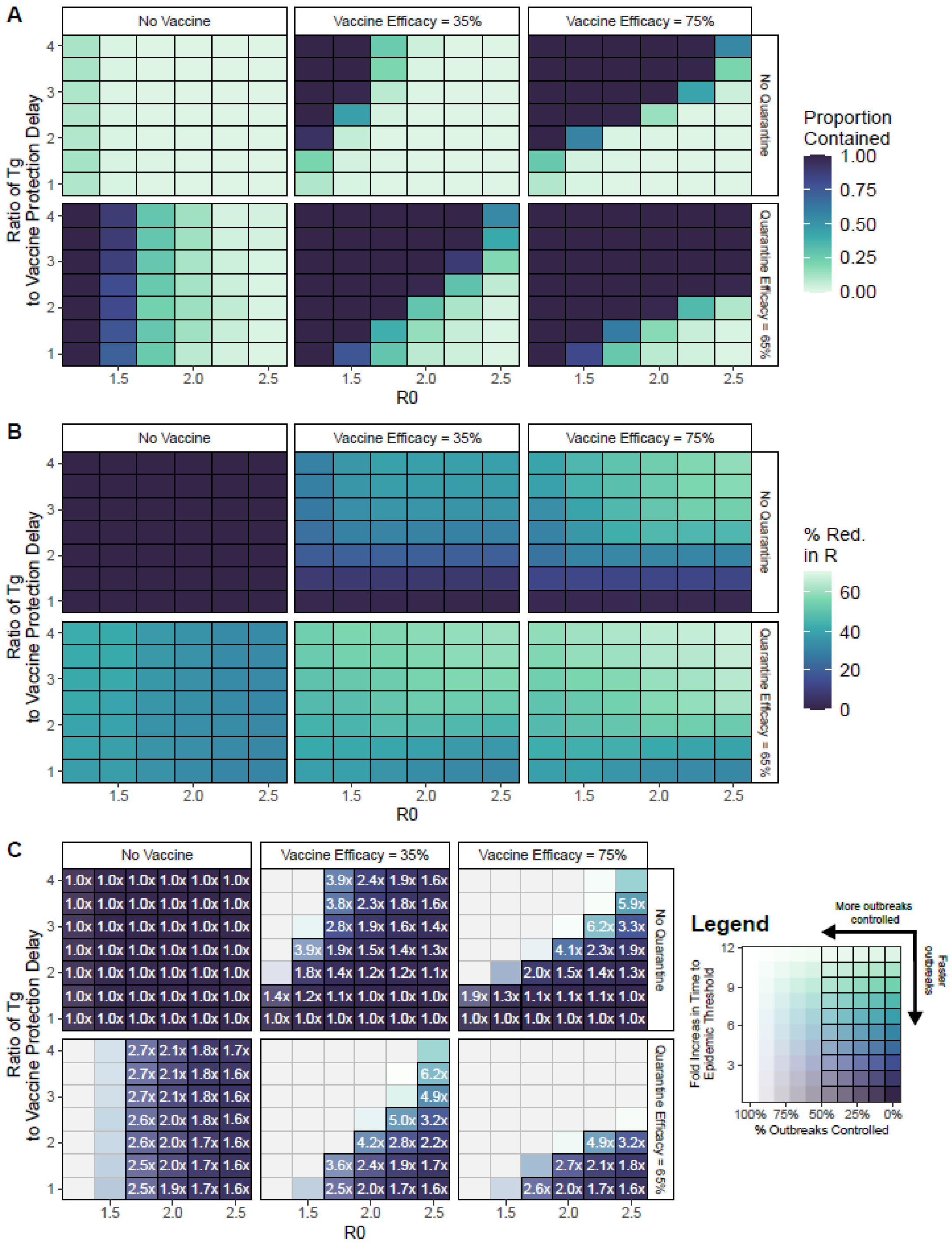

Figure S2: Supplementary results for ring-vaccination sensitivity analyses varying R0 and the ratio of the average generation time to the vaccine protection delay. (A) The proportion of outbreaks contained whilst varying R0 and the ratio of the generation time to the vaccine protection delay. Facet rows indicate assumptions about the efficacy of quarantine/isolation that occurs in response to symptoms developing; facet columns indicate assumed vaccine efficacy against infection. **(B)** As for **(A)** but plotting the average percentage reduction in the reproduction number (R) for each strategy and parameter combination. **(C)** As for **(A)** and **(B)** but plotting the time taken to reach the epidemic threshold in contexts where the outbreak was not successfully controlled. Colour (and white text inside each tile) indicates the time taken to reach the epidemic threshold relative to a situation with no vaccine, transparency indicates the percentage of outbreaks successfully controlled. For ease of visualisation, white text is only included for scenarios where <50% of epidemics were successfully controlled; these scenarios are highlighted with a black border.

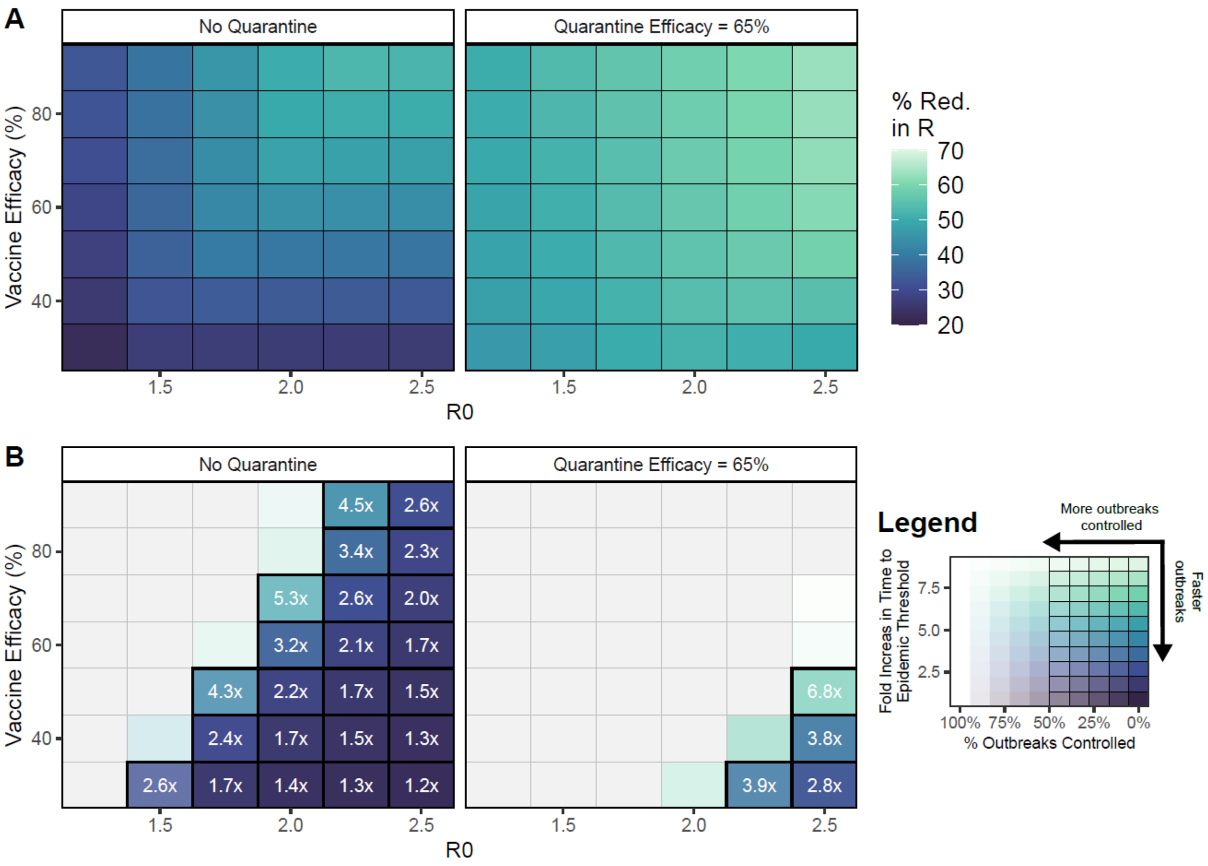

Figure S3: Supplementary results for ring-vaccination sensitivity analyses varying R0 and vaccine efficacy against infection. (A) The reduction in R achieved by ring-vaccination whilst varying R0 and the assumed efficacy of the BPSV. Facet columns indicate assumptions about the efficacy of quarantine/isolation that occurs in response to symptoms developing. **(B)** As for **(A)** but plotting the time taken to reach the epidemic threshold in contexts where the outbreak was not successfully controlled. Colour (and white text inside each tile) indicates the time taken to reach the epidemic threshold relative to a situation with no vaccine, transparency indicates the percentage of outbreaks successfully controlled. For ease of visualisation, white text is only included for scenarios where <50% of epidemics were successfully controlled; these scenarios are highlighted with a black border.

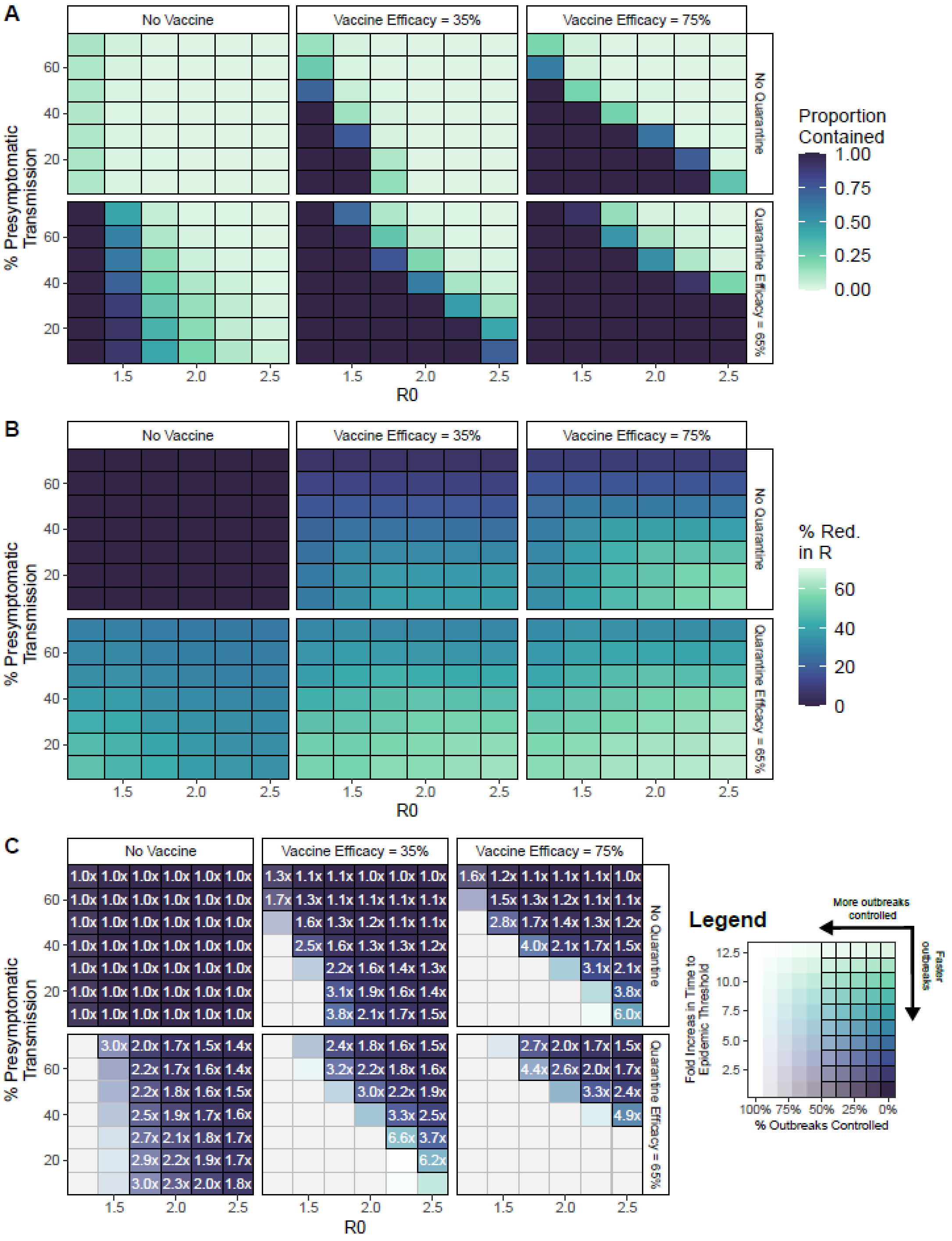

Figure S4: Supplementary results for ring-vaccination sensitivity analyses varying R0 and the extent of pre-symptomatic transmission. (A) The proportion of outbreaks contained whilst varying R0 and the fraction of transmission that is pre-symptomatic (i.e. occurs before symptoms occur). Facet rows indicate assumptions about the efficacy of quarantine/isolation that occurs in response to symptoms developing; facet columns indicate assumed vaccine efficacy against infection. **(B)** As for **(A)** but plotting the average percentage reduction in the reproduction number (R) for each strategy and parameter combination. **(C)** As for **(A)** and **(B)** but plotting the time taken to reach the epidemic threshold in contexts where the outbreak was not successfully controlled. Colour (and white text inside each tile) indicates the time taken to reach the epidemic threshold relative to a situation with no vaccine, transparency indicates the percentage of outbreaks successfully controlled. For ease of visualisation, white text is only included for scenarios where <50% of epidemics were successfully controlled; these scenarios are highlighted with a black border.

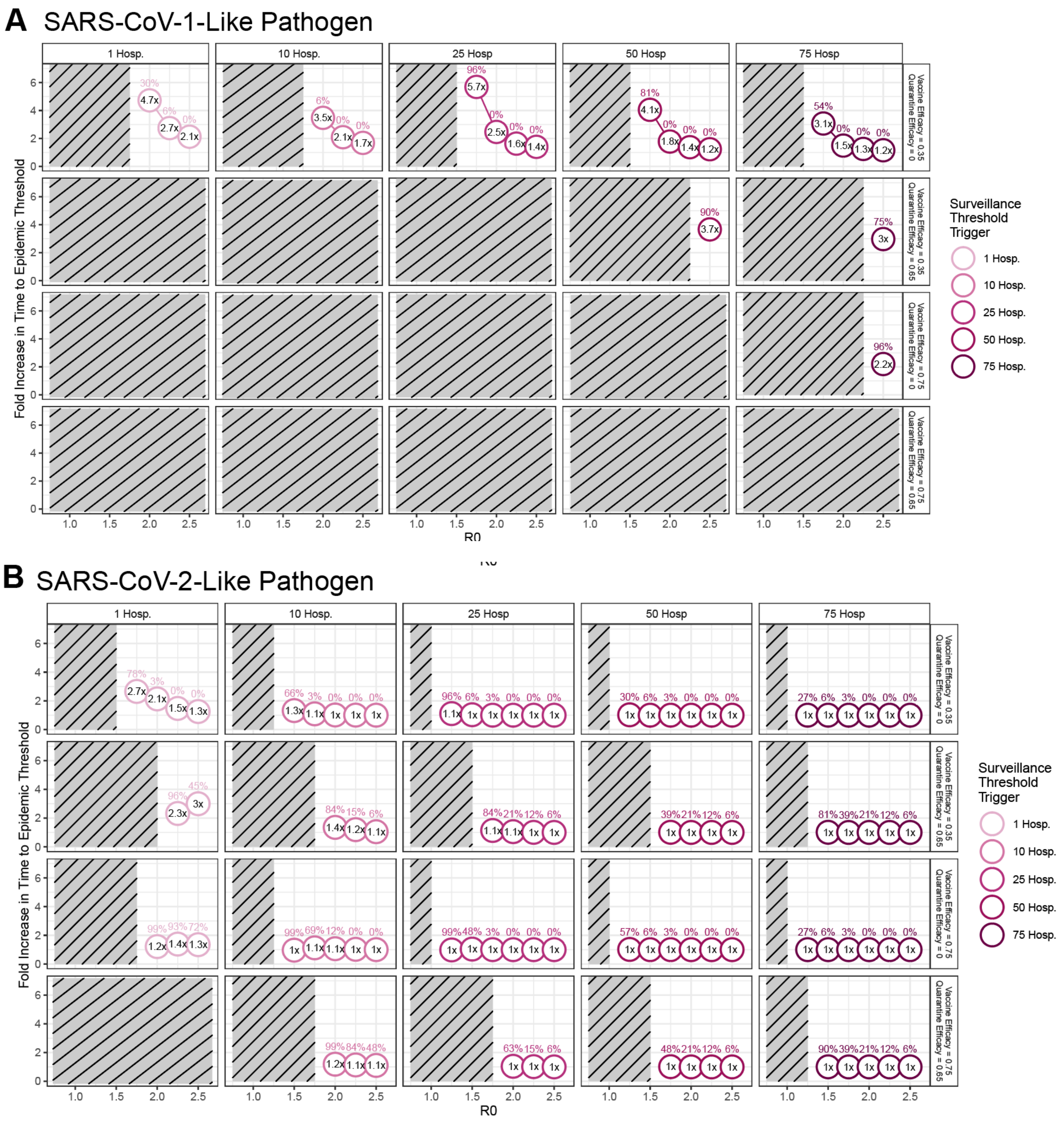

Figure S5: Time to reach the epidemic threshold in uncontrolled epidemics across different spatially targeted vaccination scenarios (A) Time to reach the epidemic threshold (10,000 infections) for the "SARS-CoV-1-Like" pathogen archetype relative to a no vaccination scenario. Facet columns (and colour) indicate the assumed trigger (number of hospitalisations) required for initiation of the spatially targeted vaccination campaign.Facet rows the combination of vaccine efficacy against infection (either 35% or 75%) and quarantine/isolation effectiveness (either no quarantine or 65% efficacy). Black text inside each point denotes the fold increase in time to reach 10,000 infections relative to a no vaccination scenario; coloured text above each point indicates the % of outbreaks that are controlled successfully i.e. 0% means no outbreaks were successfully controlled (limited to <10,000 infections). Greyed out area with black lines indicates scenarios where 100% of outbreaks were successfully controlled. Results are based on 100 stochastic simulations, with the mean fold time to increase for unsuccessfully controlled outbreaks plotted inside the circle. **(B)** As for **(A)**, but for the "SARS-CoV-2-Like" pathogen archetype.

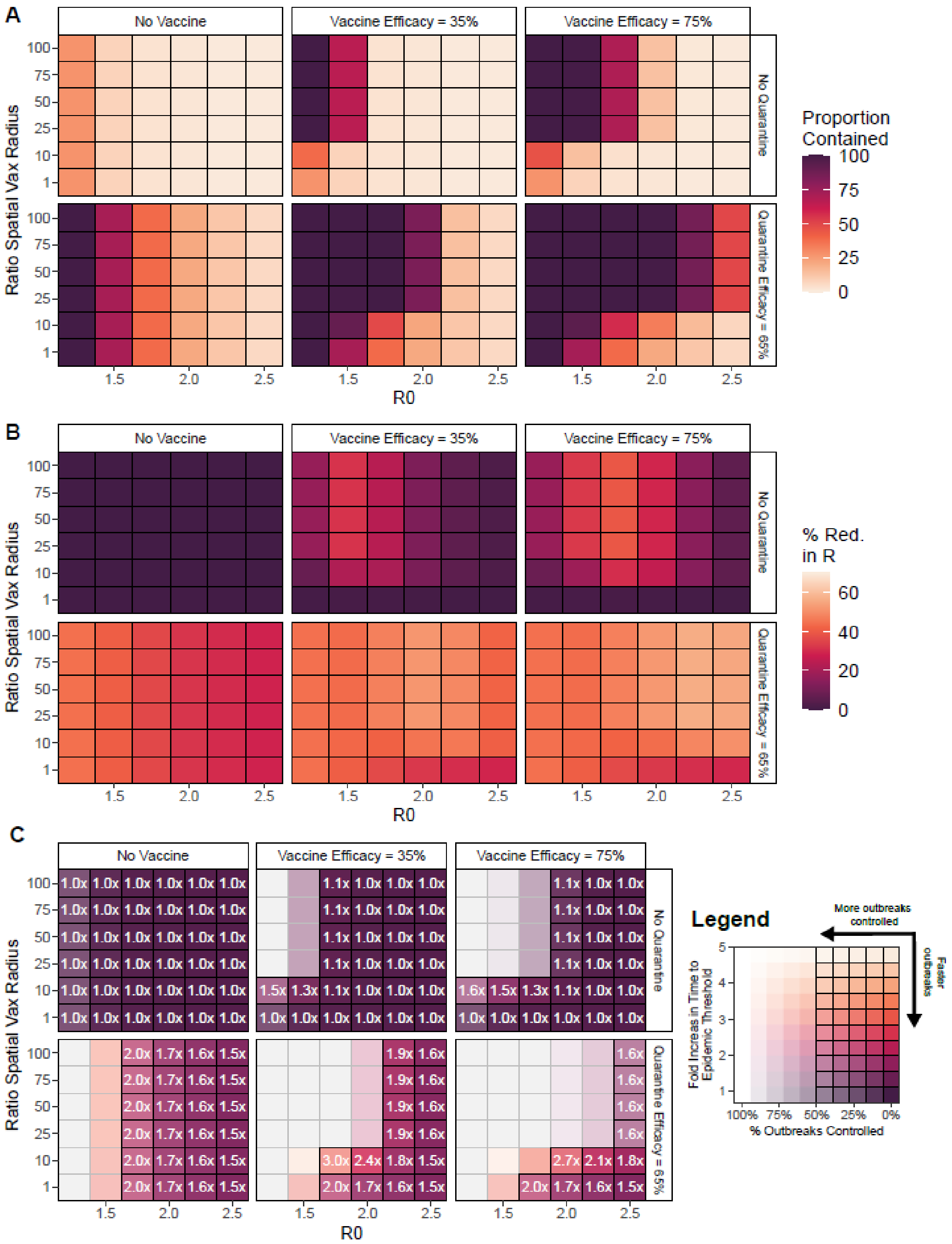

Figure S6: Supplementary results for spatially targeted vaccination strategy sensitivity analyses varying R0 and the ratio of the average distance between infections and the size of the spatial campaign. (A) The proportion of outbreaks contained whilst varying R0 and the ratio of the average distance between infections to the size of vaccination campaign (a higher number indicates a campaign spanning a larger geographical area). Facet rows indicate assumptions about the efficacy of quarantine/isolation that occurs in response to symptoms developing; facet columns indicate assumed vaccine efficacy against infection. **(B)** As for **(A)** but plotting the average percentage reduction in the basic reproduction number (R0) for each strategy and parameter combination. **(C)** As for **(A)** and **(B)** but plotting the time taken to reach the epidemic threshold in contexts where the outbreak was not successfully controlled. Colour (and white text inside each tile) indicates the time taken to reach the epidemic threshold relative to a situation with no vaccine, transparency indicates the percentage of outbreaks successfully controlled. For ease of visualisation, white text is only included for scenarios where <50% of epidemics were successfully controlled; these scenarios are highlighted with a black border.

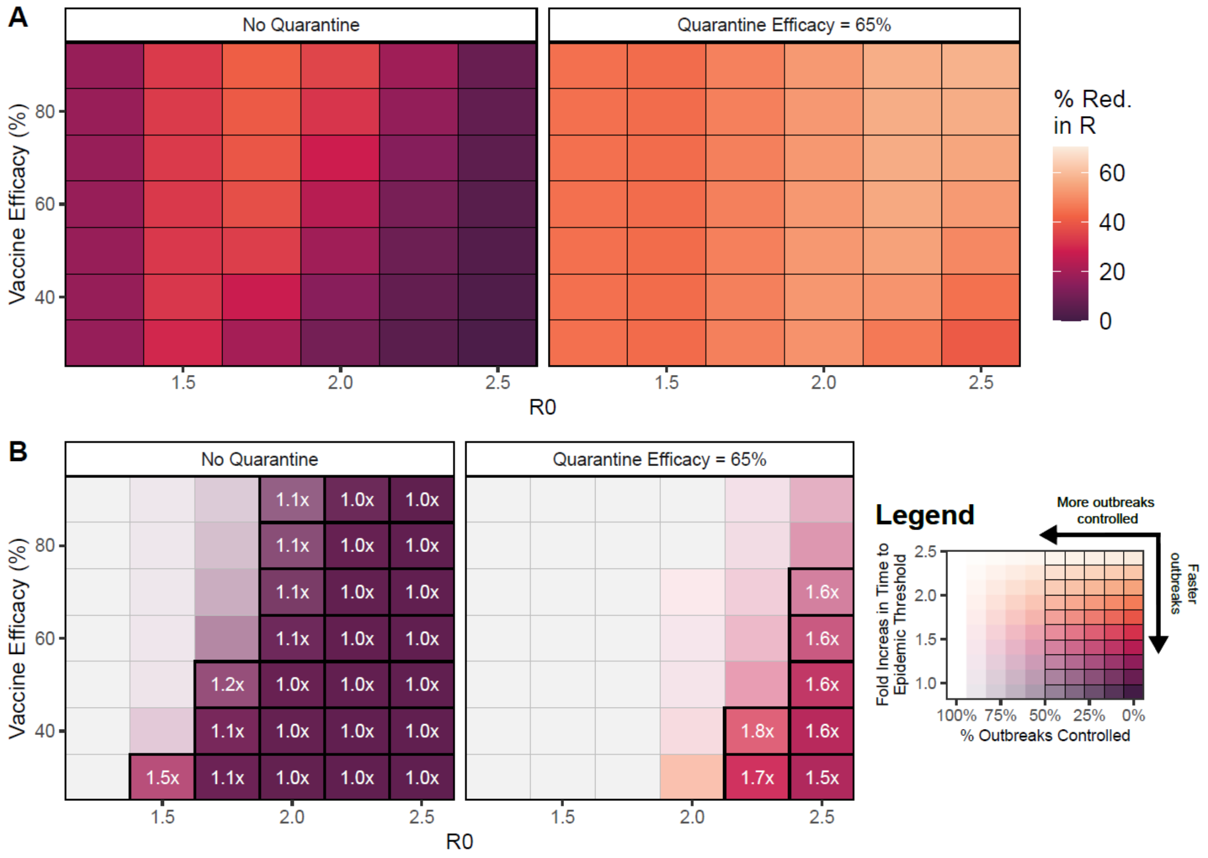

Figure S7: Supplementary results for spatially targeted vaccination sensitivity analyses varying R0 and vaccine efficacy against infection. (A) The proportion of outbreaks contained whilst varying R0 and the assumed efficacy of the BPSV. Facet rows indicate assumptions about the efficacy of quarantine/isolation that occurs in response to symptoms developing; facet columns indicate assumed vaccine efficacy against infection. **(B)** As for **(A)** but plotting the time taken to reach the epidemic threshold in contexts where the outbreak was not successfully controlled. Colour (and white text inside each tile) indicates the time taken to reach the epidemic threshold relative to a situation with no vaccine, transparency indicates the percentage of outbreaks successfully controlled. For ease of visualisation, white text is only included for scenarios where <50% of epidemics were successfully controlled; these scenarios are highlighted with a black border.

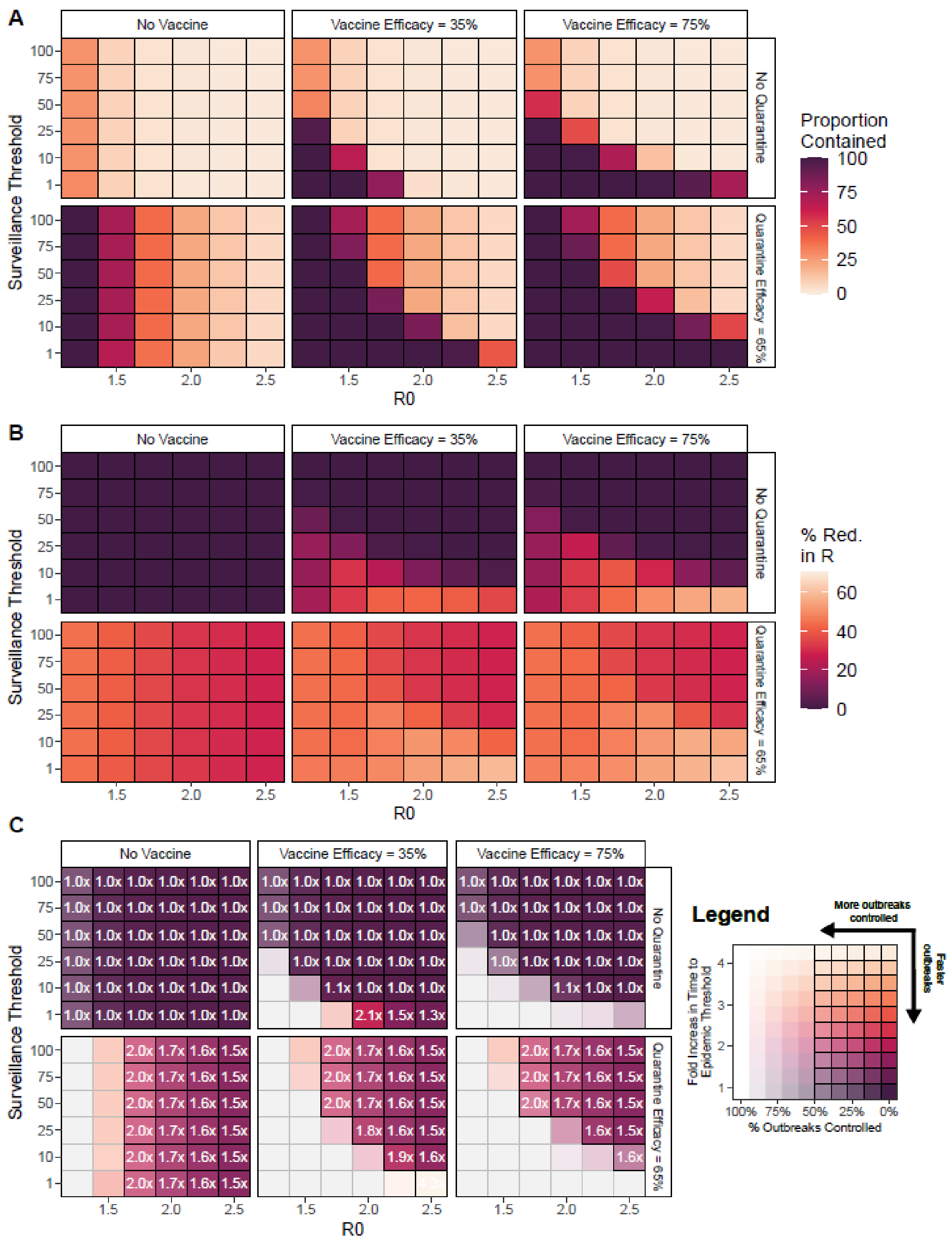

Figure S8: Supplementary results for spatially targeted vaccination strategy sensitivity analyses varying R0 and the hospitalisation threshold required to trigger the vaccination campaign. (A) The proportion of outbreaks contained whilst varying R0 and the hospitalisation threshold required to trigger the vaccination campaign. Facet rows indicate assumptions about the efficacy of quarantine/isolation that occurs in response to symptoms developing; facet columns indicate assumed vaccine efficacy against infection. **(B)** As for **(A)** but plotting the average percentage reduction in the basic reproduction number (R0) for each strategy and parameter combination. **(C) (C)** As for **(A)** and **(B)** but plotting the time taken to reach the epidemic threshold in contexts where the outbreak was not successfully controlled. Colour (and white text inside each tile) indicates the time taken to reach the epidemic threshold relative to a situation with no vaccine, transparency indicates the percentage of outbreaks successfully controlled. For ease of visualisation, white text is only included for scenarios where <50% of epidemics were successfully controlled; these scenarios are highlighted with a black border.

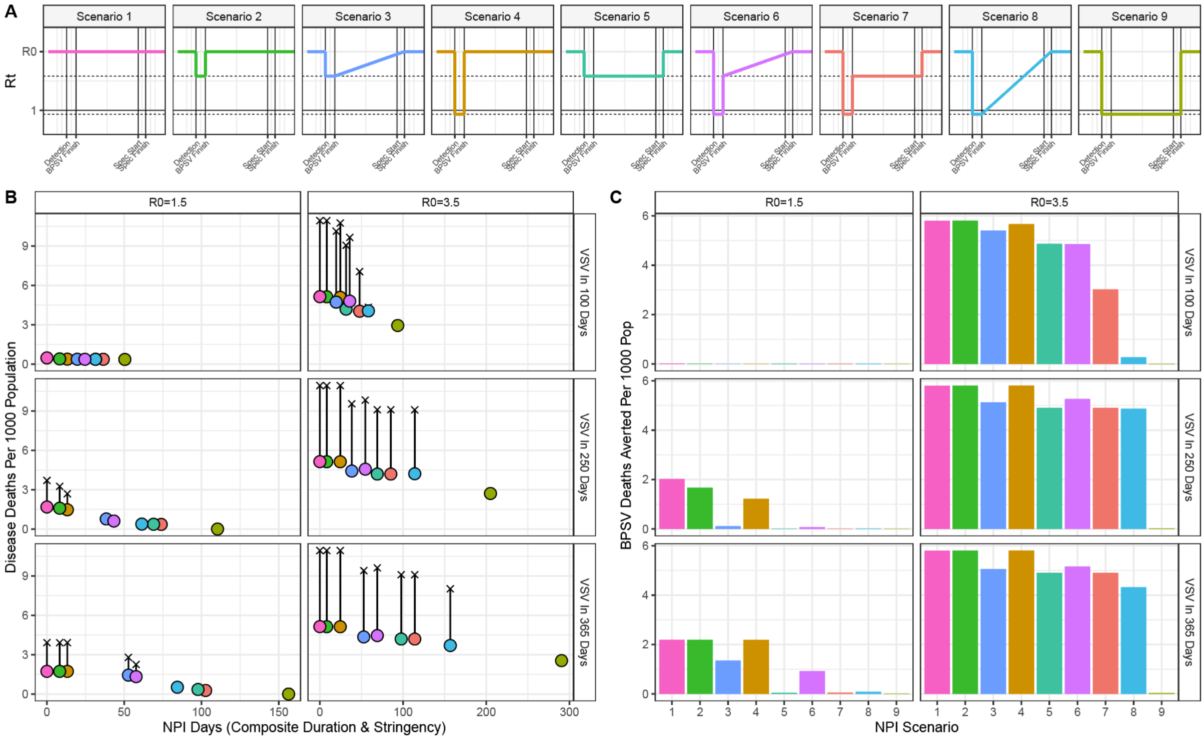

Figure S9: The influence of NPIs, R0 and VSV development timelines on BPSV impact during a SARS-X pandemic. (A) Time-varying reproduction number (Rt) profiles for the different non-pharmaceutical intervention (NPI) scenarios imposed in response to the epidemic that are considered for the analyses presented here. These Scenarios differ by assumed stringency (either no measures, a minimal mandate reducing transmission by 25% or stringent measures reducing Rt to 0.9), duration (either until the BPSV campaign is completed or the disease specific vaccination campaign is completed) and the nature by which these NPIs are relaxed (either instantaneous or gradual). **(B)** BPSV impact on disease burden for each NPI scenario, assuming the VSV is available 100 days (top-row), 250 days (middle row) or 365 days (bottom row) following detection, for an R0 of 1.5 (left hand column) or 3.5 (right hand column). Uncoloured crosses indicate scenario without BPSV (VSV only); points indicate scenarios where BPSV is available, coloured according to NPI scenario. **(C)** Deaths averted by the BPSV, coloured by NPI scenario and stratified by each unique VSV and R0 scenario considered here.

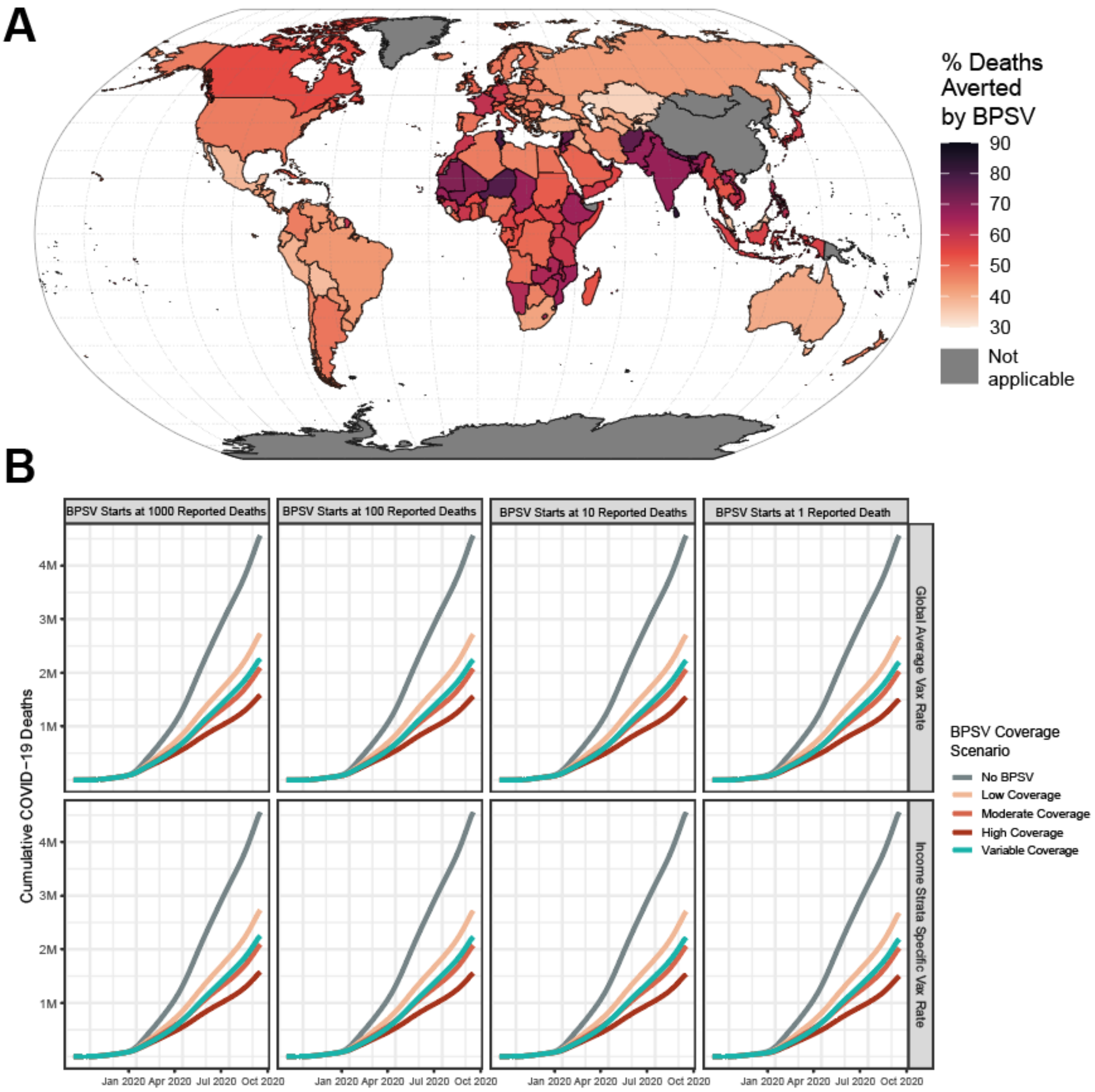

Figure S10: Assessing variation in BPSV deployment time and achieved coverage on impact. **(A)** Modelled impact of the BPSV during the first year of the COVID-19 pandemic in different countries around the world, assuming stockpile size sufficient to vaccinate 60% of each country's eligible population ("Moderate coverage" scenario). Country colour indicates the percentage of COVID-19 deaths occurring in the first year of the pandemic that could have been averted if a BPSV had been available. Results plotted are the mean of 100 simulations, each using a different posterior Rt draw for each country, with country colour indicating the percentage of COVID-19 deaths occurring in the first year of the pandemic that could have been averted if a BPSV had been available. **(B)** Cumulative global COVID-19 deaths during the first year of the pandemic without (grey) the BPSV, and with the BPSV (coloured lines). Low coverage = BPSV stockpile size sufficient to vaccinate 40% of elderly population; Moderate coverage = 60%; High coverage = 80%. Variable coverage indicates size of stockpile varies according to the World Bank Income Group each country belongs to (LIC = 20%, LMIC = 40%, UMIC = 60%, HIC = 80%). Facet columns indicate the number of globally reported COVID-19 deaths required for activation of BPSV stockpiles and initiation of eligible population vaccination. Facet rows indicate whether a uniform vaccination rate is assumed across countries (top row) or whether an income-strata specific vaccination rate is used (bottom row).

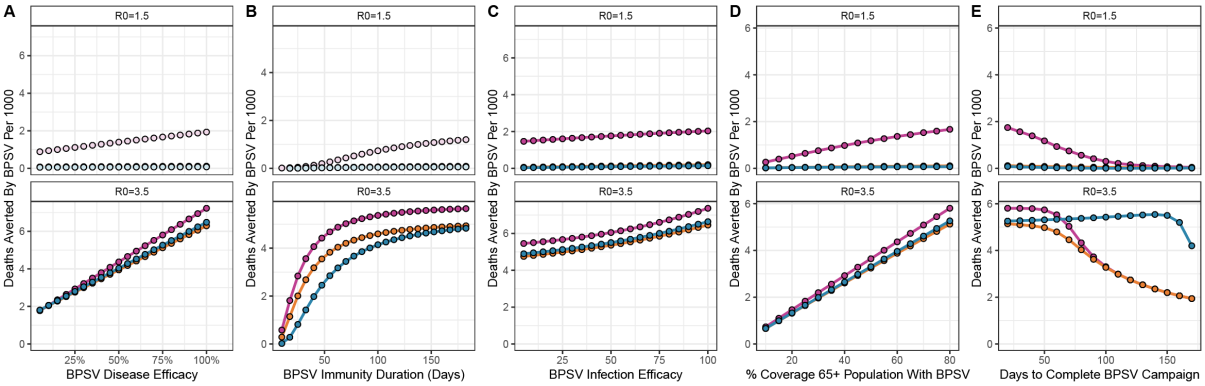

Figure S11: Dependency of BPSV impact on intrinsic vaccine properties, vaccination campaign dynamics and virus properties.

Sensitivity analyses exploring the sensitivity of BPSV impact to intrinsic BPSV properties and factors governing the speed, availability and coverage of the BPSV vaccination campaign, as well as the basic reproduction number of the virus. **(A)** Deaths averted by the BPSV (per 1,000 population) and BPSV efficacy against severe disease. Results coloured according to NPI scenario considered (pink = minimal, orange = moderate, blue = stringent), for R0=1.5 (top row) and R0=3.5 (bottom row). **(B)** As for (A) but for BPSV efficacy against infection. **(C)** As for (A) but for BPSV immunity duration. **(D)** As for (A) but for BPSV stockpile size (and associated coverage of the target population that can be achieved). **(E)** As for **(A)**, but for the rate of vaccination during the BPSV campaign (and the associated time taken to vaccinate all eligible and willing individuals).

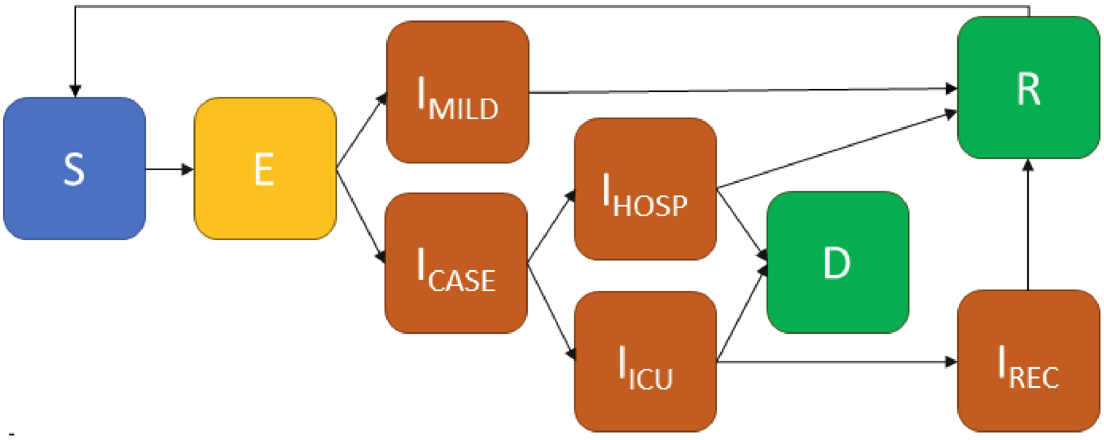

Figure S12: Model diagram of transmission, disease, and healthcare. Compartments are defined as: S - Susceptibles; E - Exposed (Latent Infection); I_{Mild} - Mild Infections (Not Requiring Hospitalisation); I_{Case} - Infections Requiring Hospitalisation; $I_{Hospital}$ - Hospitalised (Requires Hospital Bed); I_{ICU} - ICU (Requires ICU Bed); I_{Rec} - Recovering from ICU Stay (Requires Hospital Bed); R - Recovered (Acquired Immunity); D – Dead

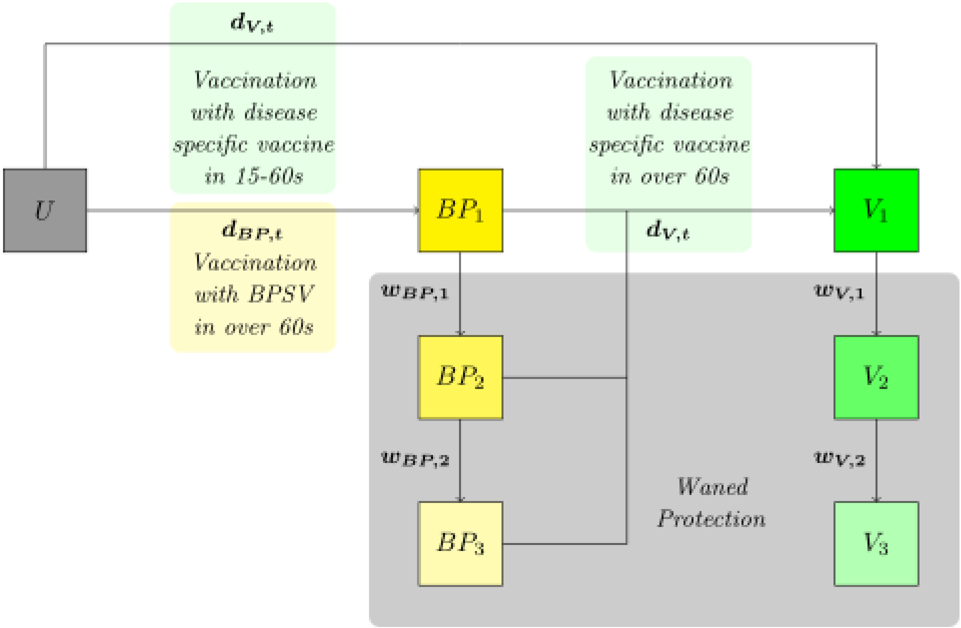

Figure S13: Model diagram of the vaccination pathway. *U* indicates those unvaccinated, ***BP*** indicates the compartments that represent those vaccinated with the BSPV vaccine, ***V*** indicates those vaccinated with the disease specific vaccine. $d_{BP,t}$ is the rate of vaccination for the BPSV and $d_{V,t}$ is the rate of vaccination for the disease specific vaccine, for both those vaccinated with the BPSV (i.e. >60s) and those unvaccinated (15-60 year olds). ω indicates the waning rates for each of the vaccine types.

References:

1. J. Hellewell, S. Abbott, A. Gimma, N. I. Bosse, C. I. Jarvis, T. W. Russell, J. D. Munday, A. J. Kucharski, W. J. Edmunds, S. Funk, R. M. Eggo, F. Sun, S. Flasche, B. J. Quilty, N. Davies, Y. Liu, S. Clifford, P. Klepac, M. Jit, C. Diamond, H. Gibbs, K. van Zandvoort, Feasibility of controlling COVID-19 outbreaks by isolation of cases and contacts. *Lancet Glob. Health* **8**, e488–e496 (2020).

2. Sebastian Funk, Flavio Finger, James M. Azam, bpmodels: Analysing transmission chain statistics using branching process models (2023) (available at https://github.com/epiverse-trace/bpmodels/).

3. A. J. Kucharski, P. Klepac, A. J. K. Conlan, S. M. Kissler, M. L. Tang, H. Fry, J. R. Gog, W. J. Edmunds, CMMID COVID-19 working group, Effectiveness of isolation, testing, contact tracing, and physical distancing on reducing transmission of SARS-CoV-2 in different settings: a mathematical modelling study. *Lancet Infect. Dis.* **20**, 1151–1160 (2020).

4. O. J. Watson, G. Barnsley, J. Toor, A. B. Hogan, P. Winskill, A. C. Ghani, Global impact of the first year of COVID-19 vaccination: a mathematical modelling study. *Lancet Infect. Dis.* **22**, 1293–1302 (2022).

5. Gregory Barnsley, Daniela Olivera Mesa, Alexandra B Hogan, Peter Winskill, Andrew A Torkleson, Damian G Walker, Azra Ghani, Oliver J Watson, *Impact of 100 Days Vaccination Mission on COVID-19: A Mathematical Modelling Study (Preprint)* (SSRN).

6. A. B. Hogan, P. Winskill, O. J. Watson, P. G. T. Walker, C. Whittaker, M. Baguelin, N. F. Brazeau, G. D. Charles, K. A. M. Gaythorpe, A. Hamlet, E. Knock, D. J. Laydon, J. A. Lees, A. Løchen, R. Verity, L. K. Whittles, F. Muhib, K. Hauck, N. M. Ferguson, A. C. Ghani, Within-country age-based prioritisation, global allocation, and public health impact of a vaccine against SARS-CoV-2: A mathematical modelling analysis. *Vaccine* **39**, 2995–3006 (2021).

7. P. G. T. Walker, C. Whittaker, O. J. Watson, M. Baguelin, P. Winskill, A. Hamlet, B. A. Djafaara, Z. Cucunubá, D. Olivera Mesa, W. Green, H. Thompson, S. Nayagam, K. E. C. Ainslie, S. Bhatia, S. Bhatt, A. Boonyasiri, O. Boyd, N. F. Brazeau, L. Cattarino, G. Cuomo-Dannenburg, A. Dighe, C. A. Donnelly, I. Dorigatti, S. L. van Elsland, R. FitzJohn, H. Fu, K. A. M. Gaythorpe, L. Geidelberg, N. Grassly, D. Haw, S. Hayes, W. Hinsley, N. Imai, D. Jorgensen, E. Knock, D. Laydon, S. Mishra, G. Nedjati-Gilani, L. C. Okell, H. J. Unwin, R. Verity, M. Vollmer, C. E. Walters, H. Wang, Y. Wang, X. Xi, D. G. Lalloo, N. M. Ferguson, A. C. Ghani, The impact of COVID-19 and strategies for mitigation and suppression in low- and middle-income countries. *Science* **369**, 413–422 (2020).

8. B. Pell, M. D. Johnston, P. Nelson, A data-validated temporary immunity model of COVID-19 spread in Michigan. *Math. Biosci. Eng.* **19**, 10122–10142 (2022).

9. M. Saville, J. P. Cramer, M. Downham, A. Hacker, N. Lurie, L. Van der Veken, M. Whelan, R. Hatchett, Delivering pandemic vaccines in 100 days - what will it take? *N. Engl. J. Med.* **387**, e3 (2022).

10. R. Verity, L. C. Okell, I. Dorigatti, P. Winskill, C. Whittaker, N. Imai, G. Cuomo-Dannenburg, H. Thompson, P. G. T. Walker, H. Fu, A. Dighe, J. T. Griffin, M. Baguelin, S. Bhatia, A. Boonyasiri, A. Cori, Z. Cucunubá, R. FitzJohn, K. Gaythorpe, W. Green, A. Hamlet, W. Hinsley, D. Laydon, G. Nedjati-Gilani, S. Riley, S. van Elsland, E. Volz, H. Wang, Y. Wang, X. Xi, C. A. Donnelly, A. C. Ghani, N. M. Ferguson, Estimates of the severity of coronavirus disease 2019: a model-based analysis. *Lancet Infect. Dis.* **20**, 669–677 (2020).

11. E. Mathieu, H. Ritchie, E. Ortiz-Ospina, M. Roser, J. Hasell, C. Appel, C. Giattino, L. Rodés-Guirao, A global database of COVID-19 vaccinations. *Nat. Hum. Behav.* **5**, 947–953 (2021).

12. Coalition for Epidemic Preparedness Innovations (CEPI), *Delivering Pandemic Vaccines in 100 Days what will it take?* (CEPI, 2022; https://cepi.net/wp-content/uploads/2022/11/CEPI-100-Days-Report-Digital-Version_29-11-22.pdf).

13. E. Mathieu, H. Ritchie, L. Rodés-Guirao, C. Appel, C. Giattino, J. Hasell, B. Macdonald, S. Dattani, D. Beltekian, E. Ortiz-Ospina, M. Roser, Coronavirus Pandemic (COVID-19). *Our World in Data* (2020) (available at https://ourworldindata.org/coronavirus).

14. N. M. Linton, T. Kobayashi, Y. Yang, K. Hayashi, A. R. Akhmetzhanov, S.-M. Jung, B. Yuan, R. Kinoshita, H. Nishiura, Incubation period and other epidemiological characteristics of 2019 novel Coronavirus infections with right truncation: A statistical analysis of publicly available case data. *J. Clin. Med.* **9**, 538 (2020).

15. Q. Li, X. Guan, P. Wu, X. Wang, L. Zhou, Y. Tong, R. Ren, K. S. M. Leung, E. H. Y. Lau, J. Y. Wong, X. Xing, N. Xiang, Y. Wu, C. Li, Q. Chen, D. Li, T. Liu, J. Zhao, M. Liu, W. Tu, C. Chen, L. Jin, R. Yang, Q. Wang, S. Zhou, R. Wang, H. Liu, Y. Luo, Y. Liu, G. Shao, H. Li, Z. Tao, Y. Yang, Z. Deng, B. Liu, Z. Ma, Y. Zhang, G. Shi, T. T. Y. Lam, J. T. Wu, G. F. Gao, B. J. Cowling, B. Yang, G. M. Leung, Z. Feng, Early transmission dynamics in Wuhan, China, of novel Coronavirus-infected pneumonia. *N. Engl. J. Med.* **382**, 1199–1207 (2020).

16. Q. Bi, Y. Wu, S. Mei, C. Ye, X. Zou, Z. Zhang, X. Liu, L. Wei, S. A. Truelove, T. Zhang, W. Gao, C. Cheng, X. Tang, X. Wu, Y. Wu, B. Sun, S. Huang, Y. Sun, J. Zhang, T. Ma, J. Lessler, T. Feng, Epidemiology and transmission of COVID-19 in 391 cases and 1286 of their close contacts in Shenzhen, China: a retrospective cohort study. *Lancet Infect. Dis.* **20**, 911–919 (2020).

17. C. Faes, S. Abrams, D. Van Beckhoven, G. Meyfroidt, E. Vlieghe, N. Hens, Belgian Collaborative Group on COVID-19 Hospital Surveillance, Time between symptom onset, hospitalisation and recovery or death: Statistical analysis of Belgian COVID-19 patients. *Int. J. Environ. Res. Public Health* **17**, 7560 (2020).

18. R. N. Thompson, F. A. Lovell-Read, U. Obolski, Time from symptom onset to hospitalisation of Coronavirus disease 2019 (COVID-19) cases: Implications for the proportion of transmissions from infectors with few symptoms. *J. Clin. Med.* **9**, 1297 (2020).

19. E. Sutherland, J. Headicar, P. Delong, *COVID-19) Infection Survey technical article - Office for National Statistics* (2021).

20. H. Salje, C. Tran Kiem, N. Lefrancq, N. Courtejoie, P. Bosetti, J. Paireau, A. Andronico, N. Hozé, J. Richet, C.-L. Dubost, Y. Le Strat, J. Lessler, D. Levy-Bruhl, A. Fontanet, L. Opatowski, P.-Y. Boelle, S. Cauchemez, Estimating the burden of SARS-CoV-2 in France. *Science* **369**, 208–211 (2020).

21. J. Sreevalsan-Nair, R. R. Vangimalla, P. R. Ghogale, Analysis and estimation of length of in-hospital stay using demographic data of COVID-19 recovered patients in Singapore*bioRxiv* (2020), doi:10.1101/2020.04.17.20069724.

22. N. J. L. Haw, J. Uy, K. T. L. Sy, M. R. M. Abrigo, Epidemiological profile and transmission dynamics of COVID-19 in the Philippines. *Epidemiol. Infect.* **148**, e204 (2020).

23. I. Hawryluk, T. A. Mellan, H. Hoeltgebaum, S. Mishra, R. P. Schnekenberg, C. Whittaker, H. Zhu, A. Gandy, C. A. Donnelly, S. Flaxman, S. Bhatt, Inference of COVID-19 epidemiological distributions from Brazilian hospital data. *J. R. Soc. Interface* **17**, 20200596 (2020).

24. E. Oliveira, A. Parikh, A. Lopez-Ruiz, M. Carrilo, J. Goldberg, M. Cearras, K. Fernainy, S. Andersen, L. Mercado, J. Guan, H. Zafar, P. Louzon, A. Carr, N. Baloch, R. Pratley, S. Silverstry, V. Hsu, J. Sniffen, V. Herrera, N. Finkler, ICU outcomes and survival in patients with severe COVID-19 in the largest health care system in central Florida. *PLoS One* **16**, e0249038 (2021).

25. ISARIC Clinical Characterisation Group, J. K. Baillie, J. Baruch, A. Beane, L. Blumberg, F. A. Bozza, T. Broadley, A. Burrell, G. Carson, B. W. Citarella, J. Dunning, L. Elotmani, N. G. Barrio, J.-C. Goffard, B. Goncalves, M. Hall, M. Hashmi, P. Horby, W. Jassat, C. Kartsonaki, B. K. T. Vijayaraghavan, P. K. Vecham, C. Laouenan, S. Lissauer, I. Martin-Loeches, F. Mentre, B. Morton, D. Munblit, N. A. Nekliudov, A. Nichol, D. S. Y. Ong, P. K. Panda, M. P. Jimenez, M. Petrovic, N. Ramakrishnan, G. V. Ramos, C. Roger, A. Rojek, O. Sandulescu, M. G. Semple, P. Sharma, S. Shrapnel, L. Sigfrid, B. S. L. Heng, B. C. Singh, E. Somers, A. Streinu-Cercel, F. S. Taccone, J. Wei, E.-J. Wils, X. C. Wong, K. Young, P. L. Olliaro, L. Merson, ISARIC COVID-19 Clinical Data Report issued: 27 march 2022*bioRxiv* (2020), doi:10.1101/2020.07.17.20155218.
